# Development and optimisation of ex situ portable X-ray fluorescence spectroscopy for heterogenous post-metallurgical sites

**DOI:** 10.1007/s10653-025-02574-x

**Published:** 2025-07-05

**Authors:** N. H. Marsay, S. T. Wagland, P. Campo, M. C. Alamar

**Affiliations:** Faculty of Engineering and Applied Sciences, Cranfield University, Cranfield, Bedfordshire, UK

**Keywords:** Metals, Post-metallurgical sites, Non-destructive technologies, Pre-treatment processes

## Abstract

Portable X-ray fluorescence spectroscopy (pXRF) is widely used for rapid measurement of metals in soils, however, thorough evaluation of common pre-processing methods and their effectiveness is limited. This study addresses processing methods using samples collected at a highly heterogeneous post-metallurgical site containing, basic oxygen steelmaking (BOS) slag and soil; the former being an important source of potentially toxic and valuable elements. Impact of pre-treatment processes, (sieving, drying, grinding, sample vessel, and ignition) on the accuracy of pXRF measurements was compared against reference ICP-MS measurements.Of the twelve elements detected, four showed qualitative (Cr and Fe r^2^ ≥ 0.60, RSD ≤ 30%) or quantitative (Mn and Ca r^2^ ≥ 0.70, RSD ≤ 20%) measurements for raw samples. Improving to six elements after pre-processing (Sr qualitative, and Pb, Cr, Mn, Ca, Fe quantitative). Sieving and grinding improved precision (average RSD fell by 7.17 and 8.37% respectively), while drying and grinding enhanced accuracy (average r^2^ increased by 0.03 and 0.10 respectively). This study provides the first evidence that organic matter does not significantly impact pXRF accuracy or precision (average r^2^ and RSD changed by zero and − 0.32%, respectively). The two distinct matrices (BOS slag and soil) on-site resulted in a bimodal concentration distribution and a negative correlation for Ti. Importantly, this research proposes that not all common pre-processing steps are necessary to generate high-quality data due to their negligible impact on accuracy or precision (such as incineration to remove organic matter), thereby increasing the speed and reducing the cost of data collection.

**Supplementary Information:**

The online version contains supplementary material available at 10.1007/s10653-025-02574-x.

## Introduction

Globally, demand for raw materials continues to grow at a steady pace, with the demand for Cu and Nd, as an example, expected to triple by 2045 (Zepf, 2014). However, the extraction and refinement of these resources has not been an efficient process, leaving a legacy of brownfield or post-metallurgical sites (Rizzo et al., 2015). Sites are comprised of contaminated soils, waste slag or mine spoil and can be sources of mobile pollution such as acid mine drainage (Marsay, 2018). Between 2000 and 2006, 1035 of these metal/metalloid contaminated sites were identified in the UK making up 60% of all contaminated sites identified in the UK (Environment Agency, 2016).

Most site surveys focus on nine potentially toxic elements (PTE) (As, Cd, Cr, Cu, Pb, Hg, Ni, Se, Zn) (Dinsdale, 2022), as these elements are present in 98% of such sites (Environment Agency, 2016). However, potentially valuable elements (PVE) such as Fe, Cu, Pt, Rh and rare earth elements (REE) (Zepf, 2020) are often overlooked in site surveys, despite post-metallurgical sites, such as former steel works, having concentrations up to 40% w/w Fe, 1.5% w/w Zn and 0.5% w/w Mn (Kara et al., 2022). However, with novel techniques such as bio- or chemical leaching (Kara et al., 2022), these PVE could be a source of new feedstocks for heavy industry and the circular economy, while reducing the cost of site remediation. Yet, to enable the remediation and recovery of these resources novel analytical methods are needed to replace the current method induction-coupled plasma mass spectrometry (ICP-MS), a highly accurate method, with a limit of detection (LOD) of < 0.01 mg kg^−1^ (Jenner et al., 1990), which is limited by its need for samples to undergo acid digestion (BSI, 2020) resulting in expensive site surveys that take several years to complete at complex sites (Dinsdale (2022).

As a compact hand-held device, portable XRF (pXRF) has been identified as a potential alternative which can be deployed in situ or ex situ, providing elemental concentrations without further data processing (Ravansari et al., 2020). However, pXRF has three well-known limitations. Ge et al (2005) observed that when sample moisture reaches 20% or more, reported metal readings are suppressed. Similarly, Ravansari and Lemke (2018) observed soil organic matter (SOM) interfering with X-rays. Finally, it has been well documented that pXRF can only measure a very small mass of sample (as low as 0.001 g for Al—Rostron & Ramsey, 2017) with a single scan, hence, results are especially susceptible to the sample heterogeneity (Kalnicky & Singhvi, 2001). As a result, in situ pXRF performs quite poorly with an average r^2^ of 0.59–0.43 when compared to ICP in (Ravansari et al., 2020). To overcome these challenges, air drying (Faithfull, 2002), oven drying (ASTM, 2020), homogenisation (sieving, grinding), and removal of SOM by ignition have been used as pre-treatments. The application of these ex situ measurement approaches resulted in a higher accuracy average with r^2^ of 0.72–0.94 (Ravansari et al., 2020).

Despite the widespread use of ex situ pXRF methods within the literature, their methodologies are inconsistent. In addition, there have been no studies investigating which pre-processing steps make the largest difference to pXRF accuracy. Furthermore, despite the importance of the sample matrix there has been no appraisal of pXRFs accuracy on basic oxygen steal-making (BOS) slag which is present in many highly heterogeneous post-metallurgical waste sites. This is a knowledge gap which needs addressing given the growing demand to remediate post-metallurgical sites and the potential resource recovery pXRF can enable. As such, this study aims to determine the effects of various sample pre-processing steps on the accuracy of pXRF measurements of highly heterogeneous post-metallurgical waste site samples.

## Methodology

### Sample material

**Fig. 1:**
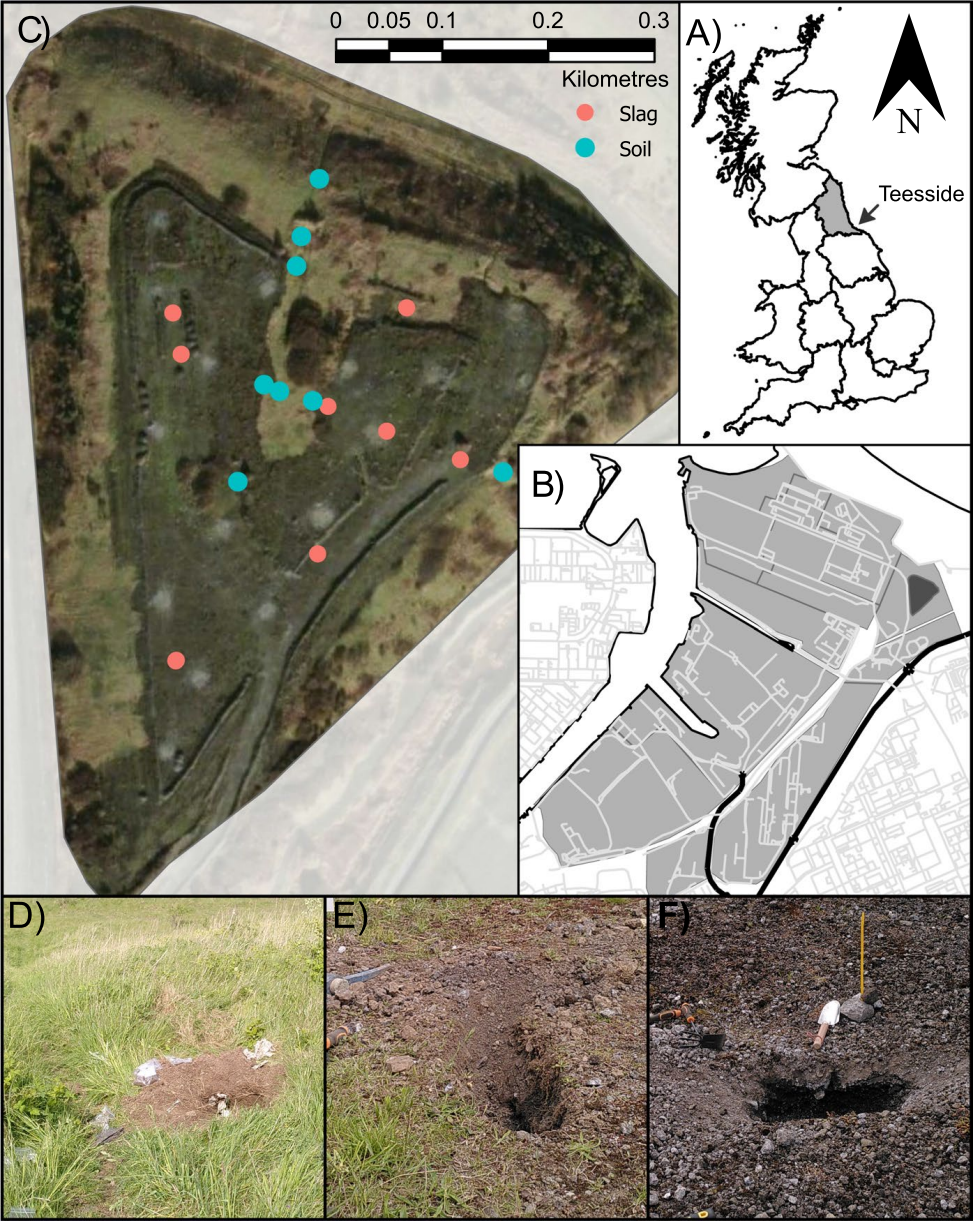
**A** Teesside (UK), and the **B** Long Acres study location (dark grey, 54.61428, − 1.10683) located within the larger Teesworks former industrial area (light grey). **C** Blue sample locations were predominately soil matrix; red sample points were predominately slag matrix. **D** Soil sample location comprised of soil with little to no slag matrix present. **E** Soil sample location with substantial slag matrix present in sample. **F** Slag sample location with no soil matrix present

Teesside, UK, is home to Europe’s largest brownfield site covering an area of over 1500 ha (South Tees Development Corporation, 2023). Comprised of the former Redcar steelworks, South Bank coke ovens, South Lackenby effluent management system and several other heavy industries and steel working sites (Fig. 1). This site has a history of heavy industry and steel refinement stretching over 160 years (Ordnance Survey, 1857). For this study, a ferric slag heap measuring 0.5 km^2^ by 18 m tall located in the Long Acers district on the east of the brownfield site was selected (Fig. 1). Documentation for the site is notably scarce. However, historical maps indicate continuous steel working activity from 1890 to 1990, with a marked increase in material disposal after the 1970s (Ordnance Survey, 1857, 1976). Available records suggest that the majority of deposited waste comprises basic oxygen steel (BOS) slag, with smaller proportions of refractory and canteen waste. Following the cessation of recorded waste disposal, the site was capped with soil. Consequently, areas with predominantly slag or predominantly soil matrices exist, with no distinct horizon separating them (Fig. 1). Locations were categorised by eye as either slag or soil based on majority of the matrix, but it should be noted that categorization was subjective and there is overlap between the grouping due to the heterogeneity of the site with some soil samples containing substantial amounts of slag (Fig. 1E). Samples were collected from the surface and sub-surface (~ 20 cm depth) of 17 locations selected to overlap with geophysical measurements (Gourry & Dumont, 2023) which captured the full range of conditions observed on-site (Fig. 1).

Where present, vegetation was cleared before sample collection. Surface samples were collected by scraping the surface with a trowel while sub-surface samples were collected from the side walls of a ~ 30 cm deep hole excavated with a pickaxe. At each location and depth, three replicates (~ 1 kg each) separated by 20 cm of distance were collected. Samples were stored in airtight polypropylene bags at room temperature for later analysis.

### Experimental design and sample preparation

**Fig. 2:**
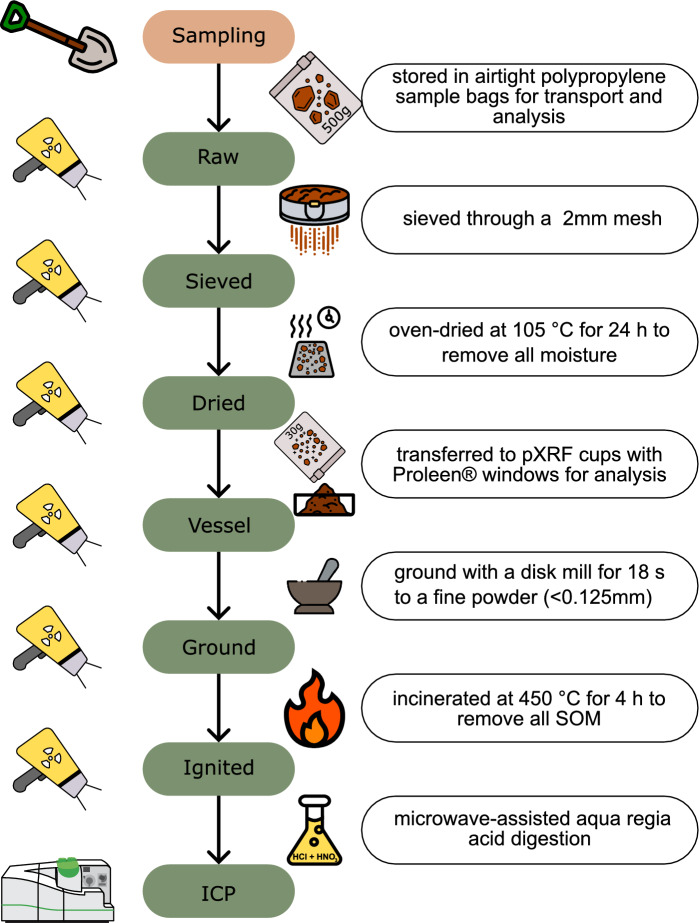
Sequential analysis and processing of samples (n = 102) collected from Teesside, UK. pXRF measurements were collected on raw, sieved, dried, vessel, ground, and ignited samples, while reference ICP-MS (induction-coupled plasma mass spectrometry) measurements were collected on acid digested samples

In sequence (Fig. 2), samples were sieved through a 2 mm mesh and the < 2 mm fraction was then dried following standard (ISO 11465:1993) procedures at 105 °C for a minimum of 24 h to remove all moisture. After drying, 30 g were carried forward to be ground with a disk mill (Siebtechnik Germany) for 18 s to a fine powder (< 0.125 mm) and further heated to 450 °C for four hours to remove all SOM in line with (BS EN 13039:2000). Finally, all samples underwent microwave-assisted acid digestion following (ISO 11047:1998). Briefly, 0.5 g of sample was dissolved in aqua regia (HCl 6 mL, HNO_3_ 2 mL, Fisher UK) followed by the standard microwaving program. After cooling, the digestate was gravity filtered and diluted 1000 times in deionised water in volumetric flasks (100 mL) and stored at room temperature until analysis.

### pXRF & ICP-MS

pXRF measurements of 26 elements (Table 1) were taken before and after each sample pre-processing step (except for acid digestion) with a Delta Premium (Olympus USA). According to manufacturer guidance, the analyser’s Rh X-ray tubes were operated in soil mode using the steel suitcase accessory; beams 1 (40 kV, Filter 3, 10 s), 2 (40 kV, Filter 1, 10 s) and 3 (15 kV, Filter 5, 30 s) were active for a total scanning time of 50 s. The first three processing steps (raw, sieved and dried) were scanned 5 times through polypropylene sample bags with shaking in-between scans; the final three stages namely, vessel (which only shows the effect of changing sample vessel), ground and ignited were scanned five times through XRF sample cups with Proleen® windows (Chemplex USA) (Figs. 1 and 2) with shaking in-between scans. A Steal 316 Alloy coin was used to perform twice daily calibration checks (EPA, 2007).

**Table 1:** Elements measured by pXRF, and ICP-MS in basic oxygen steelmaking slag samples collected from an iron slag heap in Teesside, UK

NexION 350D ICP-MS	Delta Premium pXRF
P, K, Ca, Ti, Cr, Mn, Fe, Co, Ni, Cu, Zn, As, Se, Sr, Mo, Ag Cd, Sn, Sb, Ba, Hg, Pb	P, S, Cl, K, Ca, Ti, Cr, Mn, Fe, Co, Ni, Cu, Zn, As, Se, Rb, Sr, Zr, Mo, Ag, Cd, Sn, Sb, Ba, Hg, Pb

Elements marked in bold are measured by pXRF only. LOD for pXRF and ICP-MS can be found in the supplementary information Table S1

ICP-MS measurements (Table 1) were taken by a NexION 350D ICP-MS (PerkinElmer, USA); using Scandium (Sc), Germanium (Ge), Rhodium (Rh) and Bismuth (Bi) as internal standards depending on the element analysed (Table S2). The ICP-MS was calibrated using a mixture of both major (Ca, Fe, K) and minor (P, Ti, Cr, Mn, Co, Ni, Cu, Zn, As, Se, Sr, Mo, Ag Cd, Sn, Sb, Ba, Hg, Pb) elements. Five-point linear calibration curves were created, with concentration ranges were between⁓250 and ⁓1000 μg mL^−1^ for major elements between 25 and 100 μg mL^−1^ for minor elements (Supplementary information Table S2). Limits of detection (LOD) available in supplementary information Table S1 were estimated as the concentrations corresponding to three times the standard deviation of measurements of analytes in a series of digestion blank solutions (n = 3) treated the same way as the samples. Additionally, acid blanks (1% nitric acid), digestion blank, were analysed every batch of 17–20 samples along with an adequate rinse time programmed in between samples; to monitor blank contamination, sensitivity, operating conditions, and extraction’s accuracy. All element’s concentrations have been converted into mg kg^−1^ extracted from the soil-solid matrix.

### Data analysis and quality evaluation

Data points greater or lower than the original median ± two times the interquartile range (M ± 2IQR) were considered anomalous and removed from all analyses for As, Cr, Ti, Zn, P, K, Mn, Ca, Fe and only the ICP data of Ni. However, due to their log-normal distributions Sr and pXRF data of Ni did not have values outside the M ± 2IQR removed. Due to Pb’s log-normal distribution, only the two highest values were removed from each processing step. For consistency, elements are referred to in the order of concentration measured by ICP-MS (As, Pb, Sr, Cr, Ni, Ti, Zn, P, K, Mn, Ca, Fe) in all tables, figures and within the text. Violin plots were used to show the distribution of ICP-MS measurements. Box plots were used to show the RSD distribution for each element and processing step to evaluate precision. Finally, pXRF measurements at each processing step were plotted against ICP-MS measurements for comparison purposes.

Due to the log-normal distribution, Mann–Whitney U test was used when significant differences were investigated (*p* < 0.05). Where “substantial” differences are described in this article they were visually identified in the relevant figure without the aid of a significance test. Relative standard deviation (RSD) for pXRF was calculated from the replicate scans of each sample. Mean RSD (the mean across a dataset with multiple RSD values calculated from each sample location’s replicate measurements), Pearson’s Squared correlation coefficient (r^2^), y-intercept and slope were calculated in accordance with data quality levels defined by the U.S. Environmental Protection Agency (EPA) (EPA, 1998) shown in Table 2. The author has added a “no correlation” level as the agency does not provide a lower limit to qualitative screening, but r^2^ of < 0.6 or negative slopes are ill-advised for use.

**Table 2:** US EPA levels for establishing data quality. Pearson’s Squared correlation coefficient (r^2^), relative standard deviation (RSD)

Data quality level	Statistical requirement
Definitive	r^2^ = 0.85 – 1.0. RSD ≤ 10% Inferential statistics indicate statistical similarities (At the 5% level) i.e., relationship y = x accepted
Quantitative screening	r^2^ = 0.70 – 1.0. RSD < 20% Inferential statistics indicate statistical differences i.e., relationship y = mx or y = mx + c accepted
Qualitative screening	r^2^ < 0.70. RSD 20–30% Inferential statistics indicate statistical differences
No / Unreliable correlation	r^2^ < 0.6. Negative slope. RSD > 30%

Further, goodness of fit statistics including Lin’s coordination correlation coefficient (r_c_ or ccc), ratio of performance to interquartile range (RPIQ), and root mean squared error (RMSE) can be found as supplementary material (Table S3) to aid comparison to other studies in the field. All graphs and statistical analysis were completed with the following packages in R studio v2023.09.1: tidyverse, tidymodels, scales, cowplot, patchwork and RColorBrewer (Kuhn & Wickham, 2023; Neuwirth, 2022; Pedersen, 2023; Wickham, 2023; Wickham & Seidel, 2022; Wilke, 2020). All code is available in the CORD repository (doi.org/10.57996/cran.ceres-2601).

## Results

### Descriptive statistics of ICP-MS data

In this study, samples were analysed by pXRF and ICP-MS. With pXRF, 18 elements (As, Ba, Ca, Cr, Cu, Fe, Hg, K, Mn, Ni, P, Pb, Rb, S, Sr, Ti, Zn, Zr) were detected in more than 15% of the samples. Concurrently, ICP-MS detected 12 elements (P, K, Ca, Ti, Cr, Mn, Fe, Ni, Zn, As, Sr, Pb) in more than 15% of the samples. Hence, this paper focuses on these 12 common elements, which can be compared on both instruments.

**Fig. 3:**
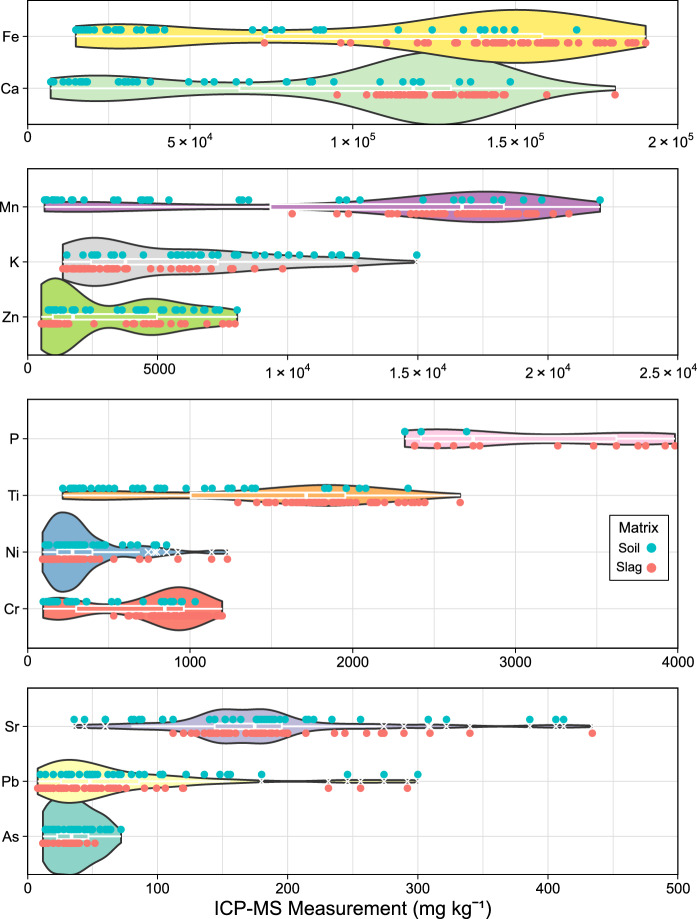
Range and distribution of all elements detected by ICP-MS in samples (n = 102) collected from an iron slag heap in Teesside, UK. Blue (soil) and red (slag) dots indicate the matrix and measurements of individual samples. Descriptive summary statistics can be found in Supplementary Table S6

Fe and Ca showed the highest concentrations on average (Fig. 3), at 115,592 and 98,875 mg kg^−1^ (11 and 9.8% w/w), respectively. Mn and K amounted to 13,518 and 5225 mg kg^−1^, respectively, followed by Zn, P, Ti, Ni, Cr, Sr, Pb, and lastly As at 35 mg kg^−1^. Additionally, XRF included three elements (Ba ~ 325, Cu ~ 25, and Hg 18 mg kg^−1^) undetected by ICP-MS.

ICP-MS results also showed bimodal distributions for Cr, Ti, P, Zn, Mn, Ca and Fe. Whereas Pb, Sr, Ni, and K had log-normal distributions. Furthermore, the bimodal distributions could be attributed to the soil and slag matrix with slag samples showing significantly higher (*p* < 0.05 Mann–Whitney U test) concentrations of Cr, Ti, P, Mn, Ca, and Fe, while Zn had significantly lower concentrations in slag (*p* < 0.05 Mann–Whitney U test) (box plot and *p* values available in supplementary information Figure S7 and Table S5).

Moisture concentrations across the sample set ranged from 0 to 22% with a median of 6% while SOM percentages ranged from 0 to 13% with a median of 3%.

### Precision

**Fig. 4:**
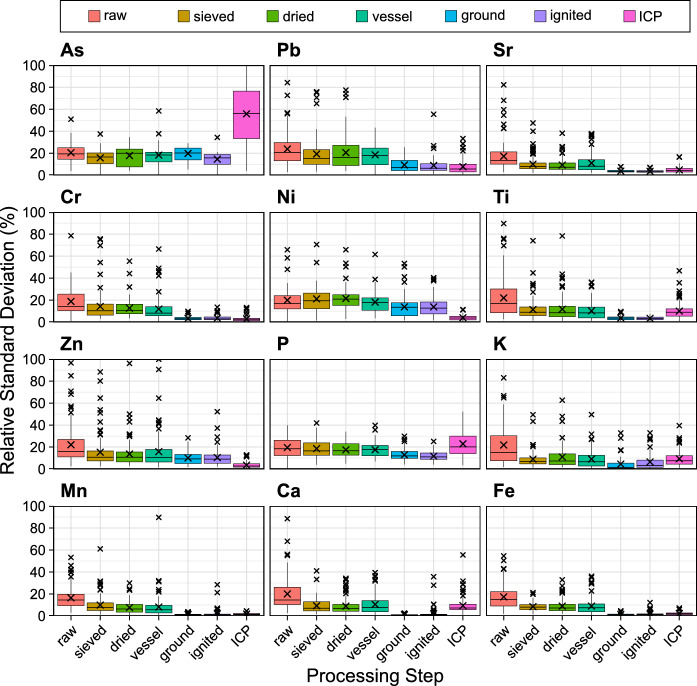
Distribution of relative standard deviations calculated across replicate scans of each sample (n = 102) collected from an iron slag heap in Teesside, UK, after each processing step measured by pXRF and after acid digestion by ICP-MS. Small crosses indicate values beyond the median ± two times the interquartile range, which were not considered anomalous due to the log-normal distribution of the dataset. The large cross indicates the mean relative standard deviation. Descriptive summary statistics of the RSD in this figure can be found in the Supplementary Table S7

To evaluate the precision of pXRF, RSD was calculated after each stage of pre-treatment from the replicate pXRF scans and the final replicate ICP-MS scans of each sample (Fig. 4). ICP-MS had very good or excellent mean RSD for all elements (< 10% EPA definitive level) except As and P whose RSD were 77% and 23%, respectively.

For raw samples, measured by pXRF six elements (Sr, Cr, Ni, P, Mn, and Fe) showed good mean RSD (< 20% EPA quantitative level). While the other six elements (As, Pb, Ti, Zn, K, Ca) showed satisfactory mean RSD (< 30% EPA qualitative level). No element showed an unsatisfactory RSD of more than 30%. Sieving made a substantial difference for eight elements (Sr, Cr, Ti, Zn, K, Mn, Ca, and Fe) reducing their mean RSD by 8% on average. Values for K, Ca, and Fe dropped below 10%, whereas Sr, Cr, Ti, Zn, and Mn resulted in < 20%.

Grinding made the largest difference to the mean RSD of nine elements (Pb, Sr, Cr, Ti, K, Mn, Ca, and Fe) decreasing by an average of 7%, whilst improving six of them (Sr, Cr, Ti, K, Mn, Ca, and Fe) to an excellent level of precision (< 5% mean RSD) and Pb to a very good precision 9.30%. Only As, Ni, and P still had good RSD after grinding (19.80, 13.86, and 12.90%, respectively). After all sample treatments As, Pb, Sr, Cr, K, Mn, and Fe were as precise as ICP-MS while As, Ti, P, and Ca were more precise than ICP.

### Accuracy

**Fig. 5:**
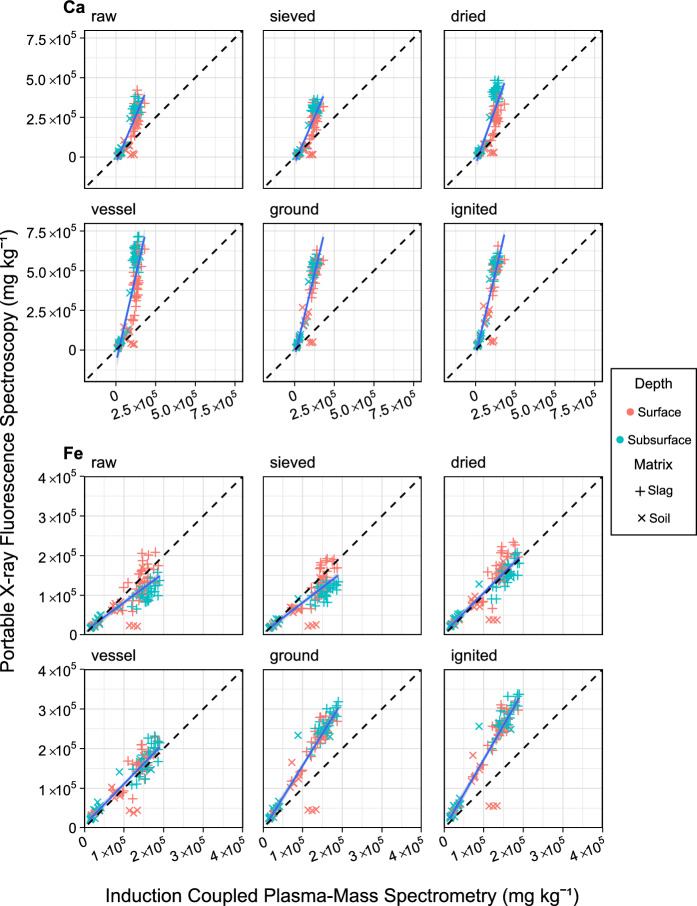
Regression of samples (n = 102) collected from an iron slag heap in Teesside, UK, showing Fe and Ca concentrations measured by pXRF after each processing step (y-axis) against ICP-MS measurements (x-axis). OLS regression (blue solid line); ideal 1–1 relationship (black dashed line); slag matrix (horizontal cross); soil matrix (diagonal cross); surface samples (red cross); subsurface samples (blue cross); standard deviation error bars can be found in supplementary Figure S4

**Fig. 6:**
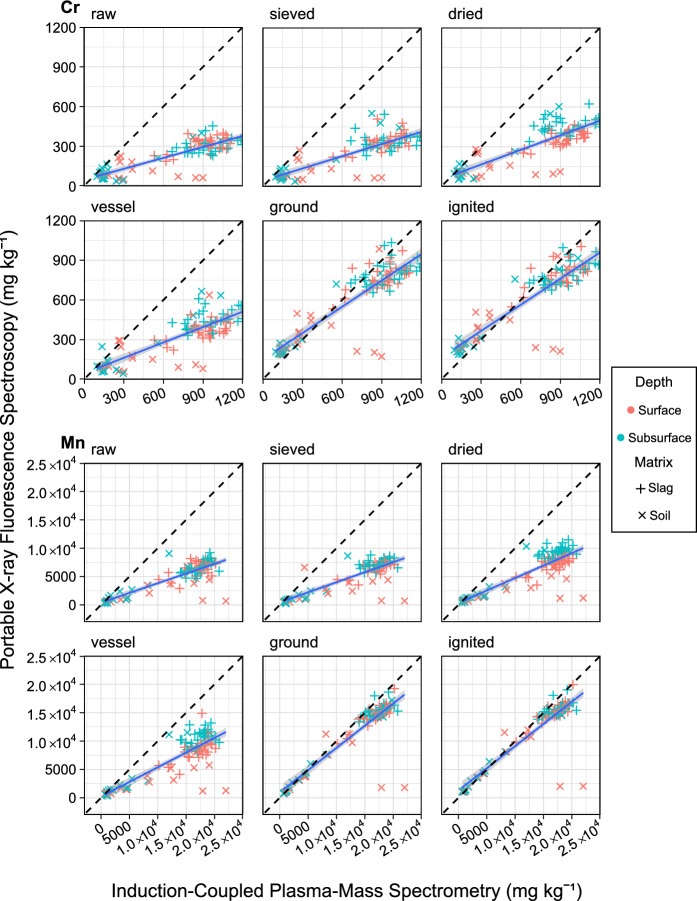
Regression of samples (n = 102) collected from an iron slag heap in Teesside, UK, showing Cr and Mn concentrations measured by pXRF after each processing step (y-axis) against ICP-MS measurements (x-axis). OLS regression (blue solid line); ideal 1–1 relationship (black dashed line); slag matrix (horizontal cross); soil matrix (diagonal cross); surface samples (red cross); subsurface samples (blue cross); standard deviation error bars can be found in supplementary Figure S3

**Fig. 7:**
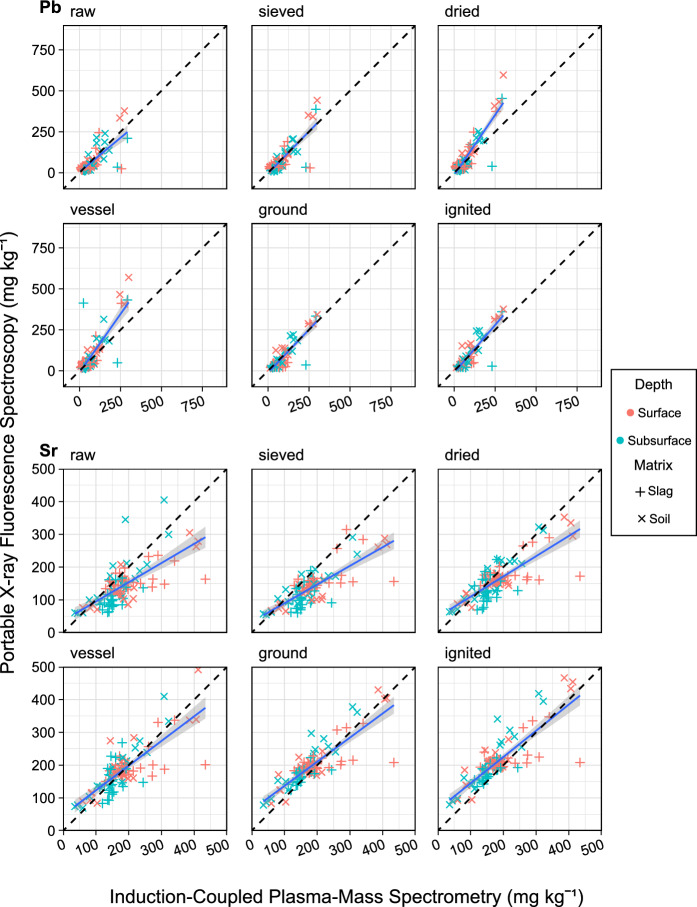
Regression of samples (n = 102) collected from an iron slag heap in Teesside, UK, showing Pb and Sr concentrations measured by pXRF after each processing step (y-axis) against ICP-MS measurements (x-axis). OLS regression (blue solid line); ideal 1–1 relationship (black dashed line); slag matrix (horizontal cross); soil matrix (diagonal cross); surface samples (red cross); subsurface samples (blue cross); standard deviation error bars can be found in supplementary Figure S4

To measure accuracy, pXRF measurements at each pre-treatment stage were plotted against ICP-MS measurements in Figs. 5, 6 and 7 and the statistical analyses required to assess performance against EPA data quality levels were presented in Table 3. Across all 12 elements, six elements Pb, Sr, Cr, Mn, Ca, and Fe (Table 3) met the qualitative (r^2^ < 0.70) or quantitative (r^2^ > 0.70) screening level as defined by the EPA while six elements As, Ni, Ti, Zn, P, and K showed no correlation (Table 3, Supplementary Tabel S3).

**Table 3:** Data quality assessment levels as defined by (EPA, 1998) and summary statistics of ordinary least squared regression on pXRF and ICP-MS measurements of samples (n = 102) from an iron slag heap

Statistic	Variable	As	Pb	Sr	Cr	Ni	Ti	Zn	P	K	Mn	Ca	Fe
n	Raw	32	86	102	99	40	101	95	57	75	100	102	102
	Sieved	33	82	99	96	44	98	92	58	69	97	99	99
	Dried	31	88	102	98	52	102	94	65	74	100	102	102
	Vessel	40	88	102	96	59	101	93	100	79	100	102	102
	Ground	21	61	102	100	97	102	90	100	52	100	102	102
	Ignited	20	56	102	100	99	102	90	100	61	100	102	102
r^2^	Raw	0.199	0.529	0.486	0.688	0.001	0.537	0.003	0.000	0.398	0.713	0.751	0.655
	Sieved	0.196	0.667	0.597	0.650	0.100	0.530	0.011	0.005	0.359	0.706	0.756	0.724
	Dried	0.251	0.802	0.612	0.689	0.005	0.594	0.005	0.021	0.390	0.713	0.682	0.800
	Vessel	0.138	0.670	0.608	0.664	0.000	0.563	0.008	0.051	0.415	0.700	0.716	0.823
	Ground	0.251	0.767	0.702	0.770	0.042	0.424	0.004	0.069	0.231	0.813	0.850	0.881
	Ignited	0.085	0.754	0.670	0.786	0.027	0.426	0.007	0.120	0.302	0.811	0.852	0.884
Mean RSD	Raw	20.5%	25.1%	18.2%	18.8%	19.9%	22.0%	22.1%	19.6%	21.8%	17.5%	20.1%	17.3%
	Sieved	15.8%	19.3%	10.0%	15.2%	21.2%	11.4%	15.3%	18.6%	9.0%	11.8%	9.3%	8.4%
	Dried	17.8%	22.0%	9.2%	12.7%	21.5%	11.8%	14.7%	17.4%	11.0%	7.6%	9.0%	9.0%
	Vessel	18.3%	19.8%	11.0%	11.9%	18.2%	10.3%	15.8%	17.6%	9.1%	8.1%	10.5%	9.0%
	Ground	19.8%	9.3%	3.7%	3.4%	13.9%	3.8%	10.1%	12.9%	4.5%	1.3%	1.0%	1.4%
	Ignited	14.6%	8.9%	3.6%	3.7%	14.8%	3.4%	10.5%	11.7%	6.5%	1.9%	2.1%	1.8%
Slope	Raw	0.154	0.832	0.587	0.275	− 0.002	− 1.005	− 0.002	− 0.043	0.650	0.342	2.361	0.740
	Sieved	0.087	1.020	0.571	0.308	0.017	− 1.046	− 0.003	− 0.208	0.629	0.354	2.325	0.768
	Dried	0.131	1.471	0.618	0.369	− 0.004	− 1.429	− 0.002	0.521	0.894	0.439	2.816	0.999
	Vessel	0.126	1.426	0.754	0.388	0.000	− 1.723	− 0.003	3.299	1.357	0.518	4.387	1.032
	Ground	0.056	1.050	0.737	0.663	0.076	− 1.059	0.001	5.449	1.026	0.783	4.171	1.614
	Ignited	0.044	1.161	0.802	0.660	0.071	− 1.154	0.002	7.238	1.268	0.782	4.211	1.734
Intercept	Raw	6	5	36	47	37	2837	134	15,629	1324	405	− 35,600	7913
	Sieved	6	− 4	33	44	29	3006	141	15,471	2029	459	− 38,360	4689
	Dried	7	− 15	48	55	42	3981	164	19,156	2102	345	− 43,470	5600
	Vessel	9	− 10	48	46	49	4885	167	26,479	2375	209	− 78,703	7489
	Ground	9	− 11	63	150	132	3916	77	39,451	3633	961	− 41,432	− 4578
	Ignited	11	− 11	65	167	149	4162	83	37,014	2043	1288	− 34,098	− 1506
Data quality	Raw	No correlation	No correlation	No correlation	Qualitative	No correlation	No correlation	No correlation	No correlation	No correlation	Quantitative	Quantitative	Qualitative
	Sieved	No correlation	Qualitative	No correlation	Qualitative	No correlation	No correlation	No correlation	No correlation	No correlation	Quantitative	Quantitative	Quantitative
	Dried	No correlation	Quantitative	Qualitative	Qualitative	No correlation	No correlation	No correlation	No correlation	No correlation	Quantitative	Qualitative	Quantitative
	Vessel	No correlation	Qualitative	Qualitative	Qualitative	No correlation	No correlation	No correlation	No correlation	No correlation	Quantitative	Quantitative	Quantitative
	Ground	No correlation	Quantitative	Quantitative	Quantitative	No correlation	No correlation	No correlation	No correlation	No correlation	Quantitative	Quantitative	Quantitative
	Ignited	No correlation	Quantitative	Qualitative	Quantitative	No correlation	No correlation	No correlation	No correlation	No correlation	Quantitative	Quantitative	Quantitative

Further statistics can be found in Supplementary Table S3

Fe performed the best with an r^2^ of 0.66 which increased to 0.88 after all processing steps. For raw and sieved samples pXRF is under-reported compared to ICP-MS. After drying, inferential statistics showed a linear relationship; grinding samples further increased r^2^ to 0.88, but it also increased the slope to 1.61, meaning that pXRF over-reported Fe compared to ICP-MS.

Ca showed a clear correlation with r^2^ of 0.75 for raw measurements rising to 0.85 after grinding. However, drying reduced r^2^ to 0.68. in addition, Ca was the only element over-predicted by pXRF when measuring raw samples, with a slope of 2.3. This was only increased by sample pre-processing, particularly the change in sample vessel, which increased the slope to 4.39.

Mn, Cr, and Sr all showed similar changes in accuracy with grinding being the only step to make a substantial change, increasing r^2^ by ~ 0.1. Similarly, grinding increased the slope of Mn and Cr by ~ 0.2, but Sr saw no substantial change in slope.

Pb, unlike the first five elements, did not show a trend of increased sample processing leading to improved r^2^ or slope. For example, drying samples increased r^2^ to 0.80 but changing sample vessels decreased r^2^ to 0.67.

Of the elements that did not show correlations, As and P had ICP-MS results with RSD which did not meet the definitive EPA level (r^2^ < 0.85) and hence their accuracy when measured by pXRF cannot be assessed in this study. Zn and Ni both had a narrow range of concentrations measured by pXRF which was < 10 and < 30% of the total range of concentrations measured by ICP-MS, respectively, resulting in slopes of ~ 0.00. K showed slopes ranging from 0.65 to 1.26 but did not reach a r^2^ higher than 0.12. Ti unexpectedly had a negative slope ranging from − 1.00 to − 1.72.

### Impact of moisture / SOM on pXRF performance

Effects of drying and igniting samples were of significant interest due to SOM and moisture being highlighted as key interferences that require further study (Ravansari et al., 2020) as such a more detailed analysis is reported below.

In the case of ignition, no substantial difference was seen in r^2^ or the slope for any element (Table 3). Drying also made no substantial difference in slope or r^2^ for nine elements (As Sr, Cr, Ni, Ti, Zn, P, K, and Mn). However, drying did substantially increase r^2^ for Fe and Pb from 0.72 to 0.80 and 0.67 to 0.80 respectively. Unexpectedly, drying decreased r^2^ for Ca from 0.76 to 0.68. Additionally, drying increased the slope of all 3 elements with Fe increasing from 0.77 to 1.00, Ca from 2.32 to 2.81, and Pb from 1.02 to 1.47.

To further investigate if moisture and SOM were important interferences, pXRF measurements of sieved/dried samples and ground/ignited were regressed against each other for moisture and SOM respectively (Table 4). Removing SOM from samples made no substantial difference to pXRF measurements with slopes for elements that met EPA data quality levels (Pb, Sr, Cr, Mn, Ca, Fe) ranging from 0.89 to 1.01 and r^2^ of > = 0.98.

**Table 4:** Regression analysis of pXRF measurements before and after **A** drying and **B** igniting samples (n = 102) collected from an iron slag heap in Teesside, UK

	Moisture			Soil organic matter		
Element	r^2^	Slope	Intercept	r^2^	Slope	Intercept
As	0.65	0.61	2.34	0.55	0.71	2.00
Pb	0.86	0.70	5.50	1.00	0.90	1.02
Sr	0.90	0.88	− 4.85	0.98	0.89	9.66
Cr	0.82	0.74	20.50	0.98	1.01	− 18.90
Ni	0.50	0.66	5.67	0.79	0.79	20.84
Ti	0.97	0.77	− 8.74	0.99	0.91	107.79
Zn	0.85	0.84	− 2.13	0.97	0.85	5.32
P	0.00	−0.02	15,281.36	0.98	0.98	−1346.72
K	0.98	0.73	249.89	0.98	0.92	248.47
Mn	0.91	0.77	388.10	1.00	1.00	−264.07
Ca	0.95	0.76	12,114.79	1.00	0.99	− 7300.37
Fe	0.94	0.79	− 1049.73	1.00	0.93	− 3113.28

Further Goodness of fit statistics, confidence intervals and regression plots are available in Supplementary Figs. S5 and S6 and Table S4

However, the removal of moisture from samples did make a substantial difference to pXRF measurements for all elements with the slope ranging from 0.74 to 0.88 and r^2^ ranging from 0.86 to 0.95 meaning pXRF measurements were consistently higher for all elements.

## Discussion

### Pre-processing step’s varying impact on pXRF performance

#### Raw measurements

This study hypothesised that raw pXRF measurements will meet the EPA quantitative screening level. Cr and Fe achieved qualitative (r^2^ < 0.70) screening levels while Mn and Ca achieved a quantitative (r^2^ > 0.70) screening levels (Table 3) for raw measurements. However, these results underperformed those found elsewhere, who measured raw samples in sample bags, as done herein, r^2^ ranged from 0.02 to 0.99 (0.62 mean) (Chen et al., 2021; Henderson et al., 2022; Horta et al., 2021; McStay et al., 2022; Spearman et al., 2022). Compared to an r^2^ range of 0.00–0.75 (0.41 mean) across the 12 elements measured in our study. This inconsistency between raw pXRF measurements and those of other studies may be due to the matrixes measured. Most studies utilising pXRF on unprocessed samples measure soil which will often have a narrower range of particle size i.e. < 2 mm. Whereas the raw slag samples had a wide range of particle sizes ranging from gravel < 4 mm to pebble < 60 mm sized material. This is consequential as larger particles will occupy more of the pXRF’s window and leave large air gaps in between, resulting in lower precision and accuracy.

While four elements met EPA data quality levels and could be assessed in a rapid field sampling program, implementing such a program would be of little benefit as samples would need to be collected and analysed ex situ to survey all other PTE and PVE.

#### Sieving and grinding

It was hypothesised that each sample processing step would result in a substantial improvement in pXRF’s correlation to ICP-MS. Both sieving and grinding made substantial reductions to the RSD of eight elements (Fig. 4). These processing steps increase homogenisation, and hence reduce random error (Kalnicky & Singhvi, 2001). Despite the EPA publishing guidance in 1998 recommending the assessment of pXRF with RSD (EPA, 1998) only five journal articles could be identified that reported mean RSD values. These values were calculated from repeat scans of certified reference materials (CRM) which would be equivalent to ground samples in our study. These studies (Delbecque et al., 2022; Hu et al., 2014; Liu et al., 2020; Rouillon & Taylor, 2016) reported RSD ranges for As 6.1–10.6%, Pb, 0.6–9.0%, Sr 8.1%, Cr 4.1–9.0%, Ni, 4.6–11.5%, Ti 0.58%, Zn 0.82–5.3%, Mn 0.8–0.9%, and Fe 0.2%. These values are broadly similar to the mean RSDs recorded in this study, except As which was substantially less precise than other studies. These steps remove variations in physical structure, grain size distribution and chemical composition. The latter being caused by the “nugget effect”, where an anomalous high-concentration chunk of analyte may randomly be placed in front of the pXRF aperture (Rostron & Ramsey, 2017), but with reduced particle size variation, the nugget effect is substantially reduced. Finally, both slope and concentration measured by pXRF where increased by grinding for Cr, Mn, and Fe (Table 3); (Peralta et al., 2020) reported similar effects for Pb and Zn attributing the difference to the exposure of elements contained within the particles.

The results from this study suggest that best practice to achieve the highest performance with pXRF includes grinding samples to increase homogeneity and precision. In addition, where particles are suspected to have differing concentrations between their interior and the exterior, grinding will be needed to obtain measurements of the entire matrix as opposed to just the surface of the matrix. However, this processing step adds substantial time and requires specialist grinding equipment due to BOS slag’s hardness. A suitable compromise of only sieving samples may be acceptable, as this brought all 12 elements within the EPA’s quantitative screening level (RSD < 20%) and 6 within the EPA’s definitive level (RSD < 10%).

#### Drying

Previous studies (Ge et al., 2005) have highlighted moisture as an important cause of interference for pXRF. In this study, drying made no substantial difference to the RSD of any element (Fig. 4). Moisture is well documented as scattering or attenuating incident and fluorescence X-rays (Ge et al., 2005); an effect that introduces systematic error and does not affect precision. When sieved and dried samples were regressed against each other showing substantial differences for all six elements with the slope ranging from 0.74 to 0.88 and r^2^ ranging from 0.86 to 0.95 (Table 4). Meaning the removal of moisture substantially increased concentrations reported by pXRF. This finding is consistent with that of (Sahraoui & Hachicha, 2017) who reported slopes of 0.67–0.94 and r^2^ of 0.96–0.98 for Pb, Cr, Mn, Ca, and Fe when regressing ex situ and dried pXRF measurements.

Most studies of moisture’s effect on pXRF have only focused on comparing pXRF measurements before and after drying samples, not comparing change in pXRF measurements to the accepted standard methods (i.e. ICP-MS) This study only found substantial differences in the regression between sieved / ICP-MS and dried / ICP-MS for Pb, Ca, and Fe (Table 3). Furthermore, only the change to pXRF measurements of Fe resulted in a higher accuracy. In the case of Pb, its slope was increased from 1.02 to 1.47 and for Ca, r^2^ decreased from 0.76 to 0.68 and its slope increased from 2.32 to 2.28. Data was complex to interpret due to contradictory results, however, results (Table 3) suggest that while removing moisture from the sample increases pXRF measurements, it does not always increase accuracy. This is most likely due to the limitations of the pXRF’s factory calibration and results could potentially be improved with a CRM calibration (Rouillon & Taylor, 2016).

It is inconclusive whether drying samples should be part of best practice when using pXRF ex situ, suggesting further work is necessary. However, comprehensive published literature highlights moisture effects, therefore, drying should be included in ex situ methodologies. It is important to note this may lead to overprediction if factory calibrations are relied upon.

#### Sample vessel

The impacts of the sample vessel were investigated in this study as pXRF manufacturers recommend that elements lighter than Ti be measured through XRF sample cups with Proleen® windows as the lower energy fluorescence X-rays of these elements are significantly attenuated by thicker sample containers (Ravansari et al., 2020). As such, changing the sample vessel made no substantial change to RSD (Fig. 4). Furthermore, the only element that showed a substantial change in slope and accuracy was Ca. This finding corroborates the previous body of literature (Ravansari et al., 2020) stating that sample cups are not necessary for the analysis of heavier elements.

#### Ignition

This study sought to assess the importance of sample pre-processing steps for the improvement of both the accuracy and precision of pXRF measurement. Ignition made no substantial difference to the RSD of any element, in accordance with previous literature (Ravansari & Lemke, 2018), as scattering or attenuating incident and fluorescence X-rays introduce systematic error and does not affect precision. This also applies to the accuracy of pXRF before and after igniting samples when compared to ICP-MS measurements (Table 3). Furthermore, no substantial difference was seen in pXRF when comparing ground and ignited samples directly against each other (Table 4). This contradicts previous studies such as (Ravansari & Lemke, 2018) who found a clear reduction in pXRF response as organic matter increased in the sample matrix. However, the paper has a limited sample size of a single CRM and used cellulose and sugar as surrogates for SOM which is not truly representative. Furthermore, that study did not acknowledge the large level of uncertainty in their measurements which makes their inference of difference due to SOM dubious.

It should also be noted that a subsequent literature review by (Ravansari et al., 2020) highlighted SOM as a key interference referring to their own and other literature. However, revaluation of their references could not identify any reports that these studies identified SOM as an interference to their measurements (Shuttleworth et al., 2014) or that their references referred back to Ravansari’s original study (Bacon et al., 2019). More recently, (Chen et al., 2021) incorporated SOM measurements into a corrective model to improve the accuracy of Pb. But the performance was negligible increasing r^2^ from 0.911 to 0.976. Furthermore, their study is limited in that its samples contained a SOM range of 0.3–3.33%, meaning that trends should not be extrapolated to cover the full range of SOM concentrations possible in samples. In addition, their study focuses solely on model creation and does not discuss the effects of SOM or other interferences present in their study.

Results in this study require further consideration as the removal of organic mass when analyte and other matrix masses are preserved, must result in a higher concentration of analyte within the sample. There are several possible explanations for this result. Firstly, to remove SOM, samples in this study were combusted at 450 °C. However, it has previously been reported that iron-rich samples can gain mass during this process due to Fe oxidising and hence gaining mass and offsetting loss of organic mass (Vandenberghe et al., 2010). But such a process would have resulted in no measured mass change and as such can be dismissed.

A second theory may be possible when considering how pXRF measures a sample. The intensity of the signal in a pXRF measurement of a sample is a product of three factors (Ravansari et al., 2020). (i) The energy of the incident and fluorescence x-rays. (ii) The absorption/scattering ability of the matrix. The first two factors together dictate the critical thickness of a sample. The final factor is (iii) the mass of the analyte within the critical thickness of the sample. As density positively correlates with absorption (Ravansari et al., 2020) and SOM contains lighter elements than the heavy elements in BOS slag, it can be assumed that the critical thickness would be reduced after removing SOM. If this is the case, then despite the increase in analyte concentration the reduced critical thickness may mean pXRF receives the same intensity of signal and hence reports the same concentration as before the removal of SOM.

The results for removing SOM in this study cast some doubt on the need for such procedures when measuring ex situ with pXRF. However, SOM did not exceed 15% in this study, thus this may not apply at higher SOM-containing samples. Further work is required to establish the effects of SOM on critical thickness and pXRF performance. Such research could involve repeating (Ravansari & Lemke, 2018) work with a wider range of samples and organic matter or better statistical analysis. In addition, modelling work may be appropriate to understand the effects at play using software such as XMI-MSIM developed by (Schoonjans et al., 2012).

### ICP-MS as a gold standard method for BOS slag

This study aimed to determine the effects of pre-treatment on the accuracy of pXRF measurements of weathered ferric slag. As such a gold standard method was needed to compare against, therefore ICP-MS was used, as is the wider practice throughout the literature (Rouillon & Taylor, 2016). Fe showed similar concentrations to BOS slag CRMs (Meeres, 2016; Meeres et al., 2022), which ranged from 18 to 13% w/w and 27–6.8% w/w in recent studies (Yildirim & Prezzi, 2011). This similarity was also observed for Mn and Ti. However, the concentrations of P, Cr, and Ca in this study were substantially lower than those in the CRMs, with Ca ranging between 31 and 38% w/w in the CRMs (Meeres, 2016; Meeres et al., 2022) and 42–21% w/w in literature (Yildirim & Prezzi, 2011). It is important to note that CRMs and samples in other studies would have been collected shortly after production and stored in controlled environments. Whereas the field site at Teesside has undergone weathering. For instance, Ca, which was produced as CaO (Yildirim & Prezzi, 2011), has several reaction pathways. Specifically, CaO reacts with CO_2_ to produce CaCO_3_ (Plane & Rollason, 2001), which can further react with H_2_CO_3_ found in acid rain, resulting in Ca(HCO_3_)_2_, a water-soluble compound. Consequently, Ca is likely to have been mobilized at Teesside, leaving the site via the watershed. The issue emerging from these findings is the need to obtain CRMs that are as representative of the site as possible. Unfortunately, there are no CRMs of weathered BOS slag currently available; and the high concentrations of Fe, Ca, Mn, etc. in the form of spinals (Rodgers et al., 2015), would have make other CRMs such as contaminated soil unrepresentative of this matrix. As such any study setting out to effectively validate its methodologies will have to create their own at great cost. However, these findings may be somewhat limited as BOS CRMs were not analysed by in-house ICP-MS. Therefore, it is not viable to exclude the possibility that these discrepancies are due to errors in the methodology of this study.

Further challenges were encountered by ICP-MS which did not detect Ba, Cu, Hg, or Sr. However, pXRF detected them at concentrations less than 326, 24, 17, and 136 mg kg^−1^, respectively. Notably, the ICP-MS’s LOD for these four elements was lower than pXRF’s measurements by 0–2 orders of magnitude. However, pXRF can overpredict concentrations in comparison to ICP-MS by an order of magnitude as is the case for Ca (Fig. 5). Thus, it is likely that the concentrations of these elements were below ICP-MS’s limit of detection.

As and P were also close to their LOD (mean As-48.76, P-3410 mg kg^−1^ LODs As-8.34, P-2220.55 mg kg^−1^). This proximity to LOD is probably the cause of ICP-MS’s relatively poor precision for As and P (Fig. 4). As it has previously been demonstrated that as the concentration of sample analyte decreases, the RSD of the ICP-MS signal will increase (Boyer et al., 1985) Due to the high concentrations of Fe and Ca, a dilution factor of 200,000 was necessary to place them within the upper range of the ICP-MS’s calibration curve. However, if the samples were rerun with reduced dilution, there might have been a possibility of detecting Ba, Cu, Hg, and Sr and increased precision for As and P. Yet, in less dilute samples, the effects of Ca, Fe, and other matrix components would become more pronounced. These effects can interfere with the measurement of lower-concentration elements. For example, Ca isotopes form ^46^Ca^16^O^1^H^+^ and ^40^Ca^16^O^1^H^+^ in ICP-MS plasma which interfere with ^63^Cu and ^65^Cu spectral lines making measurement challenging (Reed et al., 1994). Modern ICP-MS’s makes use of internal standards and mathematical correction equations (Thomas, 2013) to compensate for spectral interferences. However, the use of these tools requires skilled operators at further time and expense. These findings cast some doubt on ICP-MS viability as a gold standard method when measuring matrixes containing disparate abundances of elements such as BOS slag. As such caution should be advised in future studies.

One final limitation of ICP-MS may have been identified below, but the interpretation of these results is somewhat speculative as BOS CRMs were not analysed by both instruments to validate interpretations. A significant observation was the over-reporting of Fe by pXRF after grinding compared to ICP-MS. In contrast to earlier findings, this study reported a slope of 1.61 (Fig. 5) much higher than the 0.87 and 0.98 reported previously (Asare et al., 2020; Watanabe et al., 2021). However, one other study reported a similar slope of 1.70 (Tepanosyan et al., 2022) which unlike the previous two also measured anthropogenic waste sites. These studies and the findings reported here used aqua regia as a digestion reagent, however, significant solid material present after digestion, and it is well known that aqua regia cannot dissolve certain recalcitrant forms of Fe common in slag such as iron spinals (Rodgers et al., 2015). However, pXRF would detect all forms of Fe within the sample regardless of speciation. ICP-MS can overcome this potential limitation using hydrofluoric acid (HF) digestion. However, as many site surveyors are interested in PTE, missing recalcitrant elements is not a concern since they are not mobile or bioavailable and hence pose little risk. Furthermore, the use of hydrofluoric acid (HF) poses significant health and safety issues (Qureshi et al., 2002). However, this limitation has implications for those looking to survey and recover PVE from a site. As knowledge of recalcitrant may be useful as new extraction methods are developed such as hydrometallurgy or biohydrometallurgy (Kara et al., 2022).

This and previous studies assumed that aqua regia and ICP-MS was an accurate and gold standard method to compare pXRF performance against. However, this study shows that when applied to post-metallurgical sites with disparate elemental concentrations this assumption is incorrect. Future research on such sites should include representative CRMs as part of their validation procedure that have been created using total extraction methods. These findings may have implications for current site surveyors who use ICP-MS as their primary methodology. Although as previously stated recalcitrant elements are generally not mobile or bioavailable and hence do not require inclusion in risk analysis.

### Overall performance of pXRF on slag

This study set out to determine the effects of pre-treatment on the accuracy of pXRF measurements of weathered ferric slag. In the case of raw pXRF measurements, the mean r^2^ was 0.41 (0.64 for 6 good elements) while ignited (ex situ) was higher at 0.44 (0.79 for 6 good elements).

These results did not meet the hypothesis that pre-processed samples would meet the EPA’s definitive data quality level (r^2^ > 0.90). Furthermore, recent studies measuring soil with pXRF had substantially higher r^2^ values than results presented within this study with average r^2^ values of 0.62 for raw measurements (Chen et al., 2021; Henderson et al., 2022; Horta et al., 2021; McStay et al., 2022; Spearman et al., 2022) and 0.79 for ex situ measurements (Acquah et al., 2022; Akanchise et al., 2020; Asare et al., 2020; Delbecque et al., 2022; Horta et al., 2021; Menšík et al., 2020; Sut‐Lohmann et al., 2022; Tepanosyan et al., 2022; Watanabe et al., 2021) with maximums of 0.99 for both bag and ex situ. This difference could be due to the substantial differences in slag and soil matrixes. For example, the concentration ranges observed within the study herein (Fe max: 190,000 mg kg^−1^) (Fig. 3), were substantially higher than the maximum observed in any other pXRF study (Fe max: 71,990 mg kg^−1^ (Qu et al., 2021)). However, improved methodologies may also be the cause, several studies have made use of CRMs to create calibration curves for pXRF (Asare et al., 2020; Liu et al., 2020). A method developed by Rouillon & Taylor (2016) increased the accuracy of pXRF to the point of outperforming ICP-MS (mean recovery for Fe 99.0% and 94.1% from 5 CRMs for pXRF and ICP-MS respectively). Rouillon’s methodology was considered for our study, to improve accuracy and to validate our ICP’s accuracy, which was assumed based on previous studies. But as discussed earlier no representative CRMs of weathered BOS slag are currently available.

Despite correlations for six of the twelve elements analysed, this study found that increased sample processing did not always lead to improved correlations. As such, best practice requires that, pXRF be validated against ICP-MS at every site and sample preparation methodology used to provide correction equations (i.e., y = mx + c) as recommended by (Ravansari et al., 2020). Based on the study’s results, pXRF can only be recommended for the analysis of Pb, Sr, Cr, Mn, Ca, and Fe in BOS slag matrixes. Limiting the benefit of pXRF as a rapid analysis tool if ICP-MS analysis will need to be carried out for other elements of interest. Especially if PTE are of interest as only two (Pb, Cr) of the nine elements currently of interest to site surveyors (H. Dinsdale, personal communication, 18th August 2022) were of useful data quality.

### Bimodal distributions in concentrations

The following section explores the bimodal distribution encountered within the samples collected at Teesside and highlights the challenges in measuring such distributions. Previous studies serving waste sites often observed high heterogeneity with multiple matrixes present and a lack of clear horizons (Boudreault et al., 2010). Therefore, it was an expected finding to observe a bimodal distribution of Fe, Ca, Mn, Ti, and Cr (Fig. 3) in the data which correlates with the two matrixes found on-site. The soil matrices populated the lower end of the distributions, as expected, considering that the soils present were added to the site as a cap, rather than originating as a waste product from the BOS process (Capstick, 2020). However, Zn and P’s bimodal distribution could not be associated with the sample matrix. This discrepancy may involve leaching processes from the slags, transferring Zn or P from the slag to the surrounding soil. Although, recent research contradicts this notion, suggesting that BOS slag is a stable material that does not generate significant leachate (Richard et al., 2006).

Ti exhibited a negative correlation between pXRF and ICP-MS (slope− 1.0 to− 1.7) across all processing steps which was attributed to opposing cluster patterns around the soil and slag matrixes which can be clearly seen in supplementary Fig S1. After grinding slag samples clustered close to the 1–1 line while soil samples were significantly over reported by pXRF. this may be due to the Ti compounds present in soil having a higher resistance to digestion than those found in BOS slag. However, further analysis is required to confirm this hypothesis such as sequential extraction (Rodgers et al., 2019) to determine the speciation of Ti compounds present in the slag and soil matrixes. This inverse correlation is contrary to previous studies which have reported positive correlations as high as r^2^ 0.97, and slope 0.89 when measuring Ti CRMs (Rouillon & Taylor, 2016). However, the results presented herein are the first to be measured across multiple matrixes and show that pXRF will not perform consistently under these conditions as evidenced by Ti inverse correlation with ICP-MS; challenging the notion that pXRF will operate with uniform performance across heterogeneous waste sites. Therefore, it would be ill-advised for future surveys to rely on pXRF at such sites without total digestion and ICP-MS measurements of many samples across a variety of the matrixes present to validate pXRF results. To overcome this challenge one avenue of further research may be applying the (Rouillon & Taylor, 2016) CRM calibration method but with the addition of CRMs of differing matrixes to make such a calibration effective at heterogeneous waste sites.

## Conclusion

This study showed pXRF produced qualitative (Cr and Fe) or quantitative (Mn and Ca) measurements for raw samples which improved after pre-treatments with Sr showing qualitative, and Pb, Cr, Mn, Ca, and Fe showing quantitative pXRF measurements. Sieving and grinding improved precision, while drying and grinding enhanced accuracy, However, this study provides the first evidence that organic matter does not significantly impact pXRF accuracy. The two distinct matrixes on-site resulted in a bimodal concentration distribution and a negative correlation for Ti. Results herein highlighted the challenges of using ICP-MS for BOS slag due to elements falling below detection limits and incomplete digestion of recalcitrant Fe by aqua regia.

Although the analysis in this study was carefully controlled, this study is limited by a lack of validation for ICP-MS and by the sequential setup of sample pre-processing meaning each step must be considered in the context of previous steps. Further work is required to establish the effect of SOM on pXRF measurement given the results of this study contradict previous literature. In addition, further research should utilise CRMs of different matrices to construct a multi-matrix calibration that may improve pXRF’s performance on complex sites such as the one measured in this study.

The results herein highlight the benefits of sample pre-processing to improve the effectiveness of pXRF and suggest a recommended methodology of sieving, drying, and grinding samples to achieve the highest accuracy and precision from pXRF and to measure samples through pXRF cups if lighter elements are of interest. However, in situ results must show a correlation otherwise pre-processing will not improve pXRF’s performance. Furthermore, given the significant variance in pXRF accuracy, validation must be advised at every site. Finally, site surveyors need to measure significantly more than the six elements accurately measured by pXRF in this study, as such ICP-MS analysis will need to be performed on every sample, meaning practitioners may be best avoiding the additional analysis and expenditure of using pXRF on BOS slag sites. However, focusing solely on the six key elements, the methodology identified in this study significantly reduces both analysis time and cost enabling the recovery or remediation of contaminants at post metallurgical sites.

## Supplementary Information

Below is the link to the electronic supplementary material.

Supplementary file1 (DOCX 2304 KB)

## Data Availability

Data underlying this paper can be accessed at https://doi.org/10.57996/cran.ceres-2601.
