# Supplementary material for: Development and optimisation of ex situ portable X-ray fluorescence spectroscopy for heterogenous post-metallurgical sites
Source: Environ Geochem Health. 2025 Jul 5;47(8):298. doi: 10.1007/s10653-025-02574-x (PMC12228670; doi:10.1007/s10653-025-02574-x)

**Supplementary materials**

**Table S1.** Limits of detection for ICP-MS, and pXRF. LODs were provided by instruments based on the calibration curve of ICP-MS and the factory calibration of pXRF. ICP-MS undiluted was calculated to account for the dilution of the sample during acid digestion.

| Limits of Detection | | | |
| --- | --- | --- | --- |
| Element | ICP-MS (ug L^-1^) | ICP-MS undiluted dilution (mg kg^-1^) | pXRF (mg kg^-1^) |
| P | 0.01 | 2220.56 | 477.00 |
| S | NA | NA | 87.00 |
| Cl | NA | NA | 43.00 |
| K | 2.43 | 485.72 | 194.00 |
| Ca | 17.43 | 3486.87 | 32.00 |
| Ti | 0.13 | 25.84 | 30.00 |
| Cr | 0.06 | 12.43 | 2.30 |
| Mn | 0.06 | 11.70 | 3.70 |
| Fe | 3.02 | 604.86 | 11.00 |
| Co | 0.01 | 1.04 | 7.00 |
| Ni | 0.01 | 1.93 | 8.00 |
| Cu | 0.08 | 16.36 | 3.90 |
| Zn | 0.38 | 76.99 | 2.10 |
| As | 0.04 | 8.35 | 1.50 |
| Se | 0.72 | 144.21 | 1.00 |
| Rb | NA | NA | 1.20 |
| Sr | 0.03 | 6.28 | 0.80 |
| Zr | NA | NA | 1.60 |
| Mo | 0.02 | 3.70 | 2.30 |
| Ag | NA | NA | 11.00 |
| Cd | 0.02 | 4.86 | 13.00 |
| Sn | 0.01 | 2.91 | 20.00 |
| Sb | 0.01 | 2.66 | 22.00 |
| Ba | 0.19 | 38.82 | 4.20 |
| Hg | 0.01 | 2.79 | 3.10 |
| Pb | 0.01 | 2.45 | 2.40 |

**Table S2**. Concentrations used to calibrate ICP-MS and internal standards used for each element.

| **Elements** | **Internal standard** | |
| --- | --- | --- |
| P, K, Ca, | Sc | |
| Ti, Cr, Mn, Fe, Co, Ni, Cu, Zn, | Ge | |
| As, Se, Sr, Mo | Rh | |
| Ag, Cd, Sn, Sb, Ba, Hg, Pb | Bi | |
|  | | |
| **ICP-MS Calibration curves** | | |
| Elements | (ug L^-1^) | accounting for dilution (mg kg^-1^) |
| P, Ti, Cr, Mn, Co, Ni, Cu, Zn, As, Se, Sr, Mo, Ag Cd, Sn, Sb, Ba, Hg, Pb | 0 | 0 |
|  | 25 | 5,000 |
|  | 50 | 10,000 |
|  | 75 | 15,000 |
|  | 100 | 20,000 |
| K, Ca | 0 | 0 |
|  | 250 | 50,000 |
|  | 500 | 100,000 |
|  | 750 | 150,000 |
|  | 1,000 | 200,000 |
| Fe | 0 | 0 |
|  | 275 | 55,000 |
|  | 555 | 111,000 |
|  | 825 | 165,000 |
|  | 1,100 | 220,000 |

**Table S3.** Additional summary statistics of ordinary least squared regression on pXRF and ICP-MS measurements of samples (n=102). n is the number of samples above pXRF and ICP-MS LOD; residual prediction deviation (RPD); Person’s correlation coefficient squared (r^2^); ratio of performance to inter-quartile Distance (RPIQ); Lin’s concordance correlation coefficient (r_c_); mean absolute error (MAE); coefficient of determination (R^2^); relative standard deviation (RSD); data quality assessment levels as defined by EPA [30].

| Element | Processing Step | n | RPD | r^2^ | RMSE | RPIQ | r_c_ | MAE | R^2^ | RSD | Intercept | Slope | Intercept 95% CI | Slope 95% CI | Data Quality |
| --- | --- | --- | --- | --- | --- | --- | --- | --- | --- | --- | --- | --- | --- | --- | --- |
| As | Raw | 32 | 0.50 | 0.20 | 34.13 | 0.64 | 0.07 | 30.71 | -3.27 | 20.46 | 5.80 | 0.15 | (-1, 13) | (0.006, 0.302) | No Correlation |
|  | Sieved | 33 | 0.52 | 0.20 | 34.45 | 0.93 | 0.04 | 30.33 | -2.84 | 15.81 | 6.45 | 0.09 | (3, 10) | (0.002, 0.172) | No Correlation |
|  | Dried | 31 | 0.54 | 0.25 | 33.03 | 0.89 | 0.07 | 29.05 | -2.56 | 17.84 | 7.12 | 0.13 | (2, 12) | (0.026, 0.237) | No Correlation |
|  | Vessel | 40 | 0.57 | 0.14 | 30.28 | 1.06 | 0.07 | 25.95 | -2.25 | 18.29 | 9.27 | 0.13 | (3, 15) | (-0.006, 0.259) | No Correlation |
|  | Ground | 21 | 0.53 | 0.25 | 33.13 | 0.83 | 0.03 | 28.96 | -2.82 | 19.80 | 9.06 | 0.06 | (7, 11) | (0.001, 0.11) | No Correlation |
|  | Ignited | 20 | 0.56 | 0.09 | 30.48 | 1.05 | 0.03 | 25.97 | -2.41 | 14.57 | 11.37 | 0.04 | (8, 15) | (-0.043, 0.131) | No Correlation |
| Pb | Raw | 86 | 1.24 | 0.53 | 50.53 | 1.24 | 0.72 | 31.27 | 0.34 | 25.14 | 5.13 | 0.83 | (-12, 22) | (0.655, 1.01) | No Correlation |
|  | Sieved | 82 | 1.40 | 0.67 | 49.00 | 1.44 | 0.80 | 30.44 | 0.48 | 19.30 | -3.57 | 1.02 | (-20, 13) | (0.856, 1.185) | Qualitative |
|  | Dried | 88 | 1.10 | 0.80 | 61.12 | 1.05 | 0.78 | 36.59 | 0.16 | 22.01 | -14.95 | 1.47 | (-31, 1) | (1.31, 1.633) | **Quantitative** |
|  | Vessel | 88 | 0.89 | 0.67 | 74.98 | 0.81 | 0.69 | 39.88 | -0.28 | 19.85 | -10.32 | 1.43 | (-32, 11) | (1.203, 1.649) | Qualitative |
|  | Ground | 61 | 1.72 | 0.77 | 41.96 | 1.45 | 0.86 | 27.57 | 0.65 | 9.30 | -11.19 | 1.05 | (-29, 7) | (0.895, 1.205) | **Quantitative** |
|  | Ignited | 56 | 1.47 | 0.75 | 49.76 | 1.47 | 0.83 | 33.61 | 0.53 | 8.89 | -10.99 | 1.16 | (-33, 11) | (0.975, 1.348) | **Quantitative** |
| Sr | Raw | 102 | 1.11 | 0.49 | 65.56 | 0.79 | 0.59 | 49.80 | 0.19 | 18.17 | 36.23 | 0.59 | (12, 60) | (0.465, 0.709) | No Correlation |
|  | Sieved | 99 | 1.14 | 0.60 | 65.16 | 0.81 | 0.59 | 49.39 | 0.22 | 10.04 | 32.55 | 0.57 | (14, 51) | (0.474, 0.667) | No Correlation |
|  | Dried | 102 | 1.46 | 0.61 | 49.95 | 1.03 | 0.72 | 35.58 | 0.53 | 9.23 | 47.83 | 0.62 | (28, 67) | (0.519, 0.718) | Qualitative |
|  | Vessel | 102 | 1.53 | 0.61 | 47.57 | 1.08 | 0.78 | 33.90 | 0.57 | 10.98 | 47.63 | 0.75 | (24, 72) | (0.631, 0.876) | Qualitative |
|  | Ground | 102 | 1.71 | 0.70 | 42.69 | 1.21 | 0.81 | 30.46 | 0.65 | 3.71 | 62.92 | 0.74 | (44, 82) | (0.64, 0.835) | **Quantitative** |
|  | Ignited | 102 | 1.40 | 0.67 | 51.98 | 0.99 | 0.76 | 39.35 | 0.49 | 3.55 | 64.55 | 0.80 | (42, 87) | (0.688, 0.916) | Qualitative |
| Cr | Raw | 99 | 0.65 | 0.69 | 530.72 | 1.24 | 0.19 | 464.87 | -1.40 | 18.80 | 46.89 | 0.28 | (17, 77) | (0.237, 0.314) | Qualitative |
|  | Sieved | 96 | 0.69 | 0.65 | 509.55 | 1.33 | 0.23 | 440.69 | -1.10 | 15.17 | 43.97 | 0.31 | (7, 81) | (0.26, 0.355) | Qualitative |
|  | Dried | 98 | 0.77 | 0.69 | 455.51 | 1.46 | 0.30 | 390.59 | -0.71 | 12.67 | 55.35 | 0.37 | (15, 96) | (0.317, 0.42) | Qualitative |
|  | Vessel | 96 | 0.76 | 0.66 | 453.35 | 1.42 | 0.31 | 393.76 | -0.73 | 11.91 | 45.97 | 0.39 | (0, 92) | (0.33, 0.447) | Qualitative |
|  | Ground | 100 | 1.80 | 0.77 | 192.04 | 3.36 | 0.81 | 144.81 | 0.69 | 3.41 | 150.23 | 0.66 | (91, 210) | (0.588, 0.739) | **Quantitative** |
|  | Ignited | 100 | 1.90 | 0.79 | 182.26 | 3.54 | 0.82 | 139.21 | 0.72 | 3.67 | 167.47 | 0.66 | (111, 224) | (0.589, 0.731) | **Quantitative** |
| Ni | Raw | 40 | 0.55 | 0.00 | 439.61 | 0.50 | 0.00 | 368.57 | -2.39 | 19.93 | 37.11 | 0.00 | (26, 48) | (-0.025, 0.021) | No Correlation |
|  | Sieved | 44 | 0.55 | 0.10 | 410.73 | 0.54 | 0.01 | 348.64 | -2.47 | 21.18 | 28.59 | 0.02 | (20, 37) | (-0.002, 0.035) | No Correlation |
|  | Dried | 52 | 0.58 | 0.01 | 379.46 | 0.59 | 0.00 | 310.35 | -2.05 | 21.52 | 42.06 | 0.00 | (34, 50) | (-0.023, 0.014) | No Correlation |
|  | Vessel | 59 | 0.59 | 0.00 | 437.81 | 0.62 | 0.00 | 355.23 | -1.94 | 18.21 | 48.76 | 0.00 | (40, 58) | (-0.019, 0.018) | No Correlation |
|  | Ground | 97 | 0.78 | 0.04 | 299.70 | 0.77 | 0.08 | 198.55 | -0.66 | 13.86 | 132.28 | 0.08 | (97, 167) | (-0.007, 0.159) | No Correlation |
|  | Ignited | 99 | 0.80 | 0.03 | 290.91 | 0.76 | 0.08 | 186.91 | -0.57 | 14.83 | 149.13 | 0.07 | (110, 189) | (-0.025, 0.166) | No Correlation |
| Ti | Raw | 101 | 0.45 | 0.54 | 1475.12 | 0.67 | -0.68 | 1368.15 | -3.95 | 22.02 | 2836.97 | -1.00 | (2526, 3148) | (-1.195, -0.815) | No Correlation |
|  | Sieved | 98 | 0.44 | 0.53 | 1529.70 | 0.71 | -0.68 | 1402.84 | -4.17 | 11.43 | 3006.23 | -1.05 | (2672, 3341) | (-1.251, -0.842) | No Correlation |
|  | Dried | 102 | 0.37 | 0.59 | 1816.72 | 0.52 | -0.61 | 1551.33 | -6.56 | 11.75 | 3980.78 | -1.43 | (3589, 4373) | (-1.668, -1.19) | No Correlation |
|  | Vessel | 101 | 0.30 | 0.56 | 2218.51 | 0.44 | -0.45 | 1754.94 | -10.17 | 10.30 | 4885.29 | -1.72 | (4378, 5393) | (-2.033, -1.414) | No Correlation |
|  | Ground | 102 | 0.37 | 0.42 | 1791.06 | 0.53 | -0.41 | 1181.68 | -6.35 | 3.78 | 3916.31 | -1.06 | (3506, 4326) | (-1.31, -0.809) | No Correlation |
|  | Ignited | 102 | 0.35 | 0.43 | 1918.80 | 0.50 | -0.38 | 1246.43 | -7.44 | 3.44 | 4161.60 | -1.15 | (3717, 4606) | (-1.425, -0.882) | No Correlation |
| Zn | Raw | 95 | 0.63 | 0.00 | 3866.63 | 1.06 | 0.00 | 3015.38 | -1.56 | 22.09 | 134.28 | 0.00 | (109, 160) | (-0.008, 0.005) | No Correlation |
|  | Sieved | 92 | 0.64 | 0.01 | 3750.18 | 1.08 | 0.00 | 2880.86 | -1.46 | 15.32 | 141.12 | 0.00 | (115, 167) | (-0.01, 0.003) | No Correlation |
|  | Dried | 94 | 0.63 | 0.00 | 3841.95 | 1.06 | 0.00 | 2976.65 | -1.52 | 14.66 | 163.94 | 0.00 | (137, 191) | (-0.009, 0.005) | No Correlation |
|  | Vessel | 93 | 0.64 | 0.01 | 3827.65 | 1.06 | 0.00 | 2953.09 | -1.49 | 15.83 | 167.06 | 0.00 | (139, 195) | (-0.01, 0.004) | No Correlation |
|  | Ground | 90 | 0.64 | 0.00 | 3853.04 | 1.05 | 0.00 | 2989.87 | -1.51 | 10.06 | 77.31 | 0.00 | (63, 92) | (-0.003, 0.005) | No Correlation |
|  | Ignited | 90 | 0.64 | 0.01 | 3845.79 | 1.05 | 0.00 | 2979.57 | -1.49 | 10.49 | 83.10 | 0.00 | (66, 100) | (-0.003, 0.006) | No Correlation |
| P | Raw | 57 | 0.05 | 0.00 | 12653.65 | 0.09 | 0.00 | 12455.28 | -439.42 | 19.55 | 15628.81 | -0.04 | (9316, 21942) | (-2.079, 1.992) | No Correlation |
|  | Sieved | 58 | 0.05 | 0.01 | 12043.25 | 0.09 | 0.00 | 11916.04 | -439.46 | 18.62 | 15470.79 | -0.21 | (10203, 20739) | (-1.964, 1.549) | No Correlation |
|  | Dried | 65 | 0.03 | 0.02 | 17831.68 | 0.06 | 0.00 | 17697.17 | -873.63 | 17.45 | 19155.57 | 0.52 | (12767, 25544) | (-1.539, 2.581) | No Correlation |
|  | Vessel | 100 | 0.02 | 0.05 | 34502.26 | 0.03 | 0.00 | 33375.75 | -3207.38 | 17.56 | 26479.46 | 3.30 | (2609, 50350) | (-4.499, 11.098) | No Correlation |
|  | Ground | 100 | 0.01 | 0.07 | 54404.54 | 0.02 | 0.00 | 52900.50 | -7693.93 | 12.90 | 39451.18 | 5.45 | (4078, 74824) | (-6.013, 16.91) | No Correlation |
|  | Ignited | 100 | 0.01 | 0.12 | 57118.08 | 0.02 | 0.00 | 55726.43 | -8792.02 | 11.72 | 37013.67 | 7.24 | (3990, 70037) | (-3.551, 18.027) | No Correlation |
| K | Raw | 75 | 1.12 | 0.40 | 3101.67 | 1.69 | 0.62 | 2094.43 | 0.20 | 21.83 | 1324.43 | 0.65 | (-20, 2669) | (0.45, 0.849) | No Correlation |
|  | Sieved | 69 | 1.10 | 0.36 | 3177.33 | 1.59 | 0.60 | 2135.33 | 0.15 | 9.02 | 2028.60 | 0.63 | (514, 3543) | (0.41, 0.848) | No Correlation |
|  | Dried | 74 | 0.84 | 0.39 | 4169.31 | 1.24 | 0.55 | 3179.41 | -0.45 | 11.02 | 2102.08 | 0.89 | (201, 4003) | (0.612, 1.176) | No Correlation |
|  | Vessel | 79 | 0.48 | 0.42 | 7187.06 | 0.76 | 0.39 | 5666.95 | -3.32 | 9.09 | 2374.62 | 1.36 | (-182, 4931) | (0.968, 1.747) | No Correlation |
|  | Ground | 52 | 0.46 | 0.23 | 7324.65 | 0.76 | 0.30 | 6136.14 | -3.81 | 4.51 | 3632.63 | 1.03 | (-603, 7869) | (0.47, 1.582) | No Correlation |
|  | Ignited | 61 | 0.45 | 0.30 | 7758.42 | 0.72 | 0.34 | 6132.34 | -3.92 | 6.48 | 2042.89 | 1.27 | (-1763, 5849) | (0.736, 1.799) | No Correlation |
| Mn | Raw | 100 | 0.68 | 0.71 | 9692.21 | 0.84 | 0.24 | 8578.54 | -1.21 | 17.47 | 405.32 | 0.34 | (-278, 1089) | (0.297, 0.388) | **Quantitative** |
|  | Sieved | 97 | 0.70 | 0.71 | 9433.14 | 1.00 | 0.26 | 8336.62 | -1.06 | 11.81 | 458.72 | 0.35 | (-269, 1187) | (0.306, 0.402) | **Quantitative** |
|  | Dried | 100 | 0.78 | 0.71 | 8332.14 | 0.97 | 0.35 | 7277.00 | -0.65 | 7.64 | 344.79 | 0.44 | (-530, 1219) | (0.381, 0.497) | **Quantitative** |
|  | Vessel | 100 | 0.88 | 0.70 | 7409.55 | 1.09 | 0.44 | 6353.54 | -0.31 | 8.14 | 209.45 | 0.52 | (-853, 1271) | (0.447, 0.588) | **Quantitative** |
|  | Ground | 100 | 1.89 | 0.81 | 3466.43 | 2.36 | 0.85 | 2135.64 | 0.72 | 1.28 | 960.72 | 0.78 | (-224, 2146) | (0.705, 0.861) | **Quantitative** |
|  | Ignited | 100 | 1.98 | 0.81 | 3301.39 | 2.47 | 0.86 | 1917.49 | 0.74 | 1.93 | 1287.60 | 0.78 | (98, 2477) | (0.704, 0.861) | **Quantitative** |
| Ca | Raw | 102 | 0.35 | 0.75 | 132687.87 | 0.49 | 0.36 | 106837.19 | -7.34 | 20.05 | -35599.57 | 2.36 | (-65624, -5575) | (2.086, 2.636) | **Quantitative** |
|  | Sieved | 99 | 0.37 | 0.76 | 125474.64 | 0.55 | 0.39 | 99947.27 | -6.39 | 9.26 | -38359.54 | 2.32 | (-67745, -8974) | (2.053, 2.597) | **Quantitative** |
|  | Dried | 102 | 0.25 | 0.68 | 182437.59 | 0.36 | 0.26 | 141342.44 | -14.77 | 8.95 | -43470.33 | 2.82 | (-85923, -1018) | (2.427, 3.205) | Qualitative |
|  | Vessel | 102 | 0.14 | 0.72 | 325437.00 | 0.20 | 0.15 | 260328.75 | -49.17 | 10.50 | -78703.31 | 4.39 | (-139664, -17743) | (3.828, 4.946) | **Quantitative** |
|  | Ground | 102 | 0.14 | 0.85 | 318958.24 | 0.20 | 0.15 | 275607.52 | -47.19 | 1.01 | -41431.88 | 4.17 | (-80100, -2763) | (3.816, 4.526) | **Quantitative** |
|  | Ignited | 102 | 0.14 | 0.85 | 329506.66 | 0.20 | 0.14 | 286489.88 | -50.43 | 2.10 | -34098.08 | 4.21 | (-72876, 4680) | (3.855, 4.567) | **Quantitative** |
| Fe | Raw | 102 | 1.40 | 0.66 | 39750.62 | 2.15 | 0.74 | 30182.14 | 0.48 | 17.33 | 7913.17 | 0.74 | (-6023, 21849) | (0.631, 0.849) | Qualitative |
|  | Sieved | 99 | 1.53 | 0.72 | 36853.44 | 2.38 | 0.78 | 27176.68 | 0.57 | 8.45 | 4688.81 | 0.77 | (-7786, 17163) | (0.67, 0.865) | **Quantitative** |
|  | Dried | 102 | 1.97 | 0.80 | 28192.05 | 3.03 | 0.89 | 20204.24 | 0.74 | 8.98 | 5599.59 | 1.00 | (-7374, 18573) | (0.898, 1.1) | **Quantitative** |
|  | Vessel | 102 | 1.94 | 0.82 | 28736.13 | 2.97 | 0.88 | 21603.57 | 0.73 | 8.99 | 7488.60 | 1.03 | (-4914, 19891) | (0.935, 1.128) | **Quantitative** |
|  | Ground | 102 | 0.68 | 0.88 | 81495.87 | 1.05 | 0.60 | 71253.52 | -1.17 | 1.36 | -4578.30 | 1.61 | (-19966, 10809) | (1.494, 1.734) | **Quantitative** |
|  | Ignited | 102 | 0.56 | 0.88 | 99007.85 | 0.86 | 0.52 | 87527.17 | -2.20 | 1.76 | -1506.48 | 1.73 | (-17836, 14823) | (1.606, 1.861) | **Quantitative** |

**Table S4.** Additional summary statistics of ordinary least squared regression on pXRF measurements before and after drying / igniting of samples (n=102). n is the number of samples above pXRF LOD; residual prediction deviation (RPD); Person’s correlation coefficient squared (r^2^); ratio of performance to inter-quartile Distance (RPIQ); Lin’s concordance correlation coefficient (r_c_); mean absolute error (MAE); coefficient of determination (R^2^); relative standard deviation (RSD).

| Processing Step | element | n | RDD | r^2^ | RMSE | RPIQ | r_c_ | MAE | R^2^ | Intercept | Slope | Intercept 95% CI | Slope 95% CI |
| --- | --- | --- | --- | --- | --- | --- | --- | --- | --- | --- | --- | --- | --- |
| Drying | As | 57 | 1.090 | 0.647 | 3.2 | 1.346 | 0.589 | 2.5 | 0.132 | 2.3 | 0.611 | (0, 4) | (0.446, 0.776) |
|  | Pb | 94 | 2.174 | 0.859 | 51.4 | 1.611 | 0.867 | 24.3 | 0.786 | 5.5 | 0.698 | (-4, 15) | (0.635, 0.76) |
|  | Sr | 105 | 1.974 | 0.903 | 29.4 | 1.759 | 0.873 | 24.2 | 0.741 | -4.8 | 0.885 | (-15, 5) | (0.826, 0.943) |
|  | Cr | 106 | 1.740 | 0.822 | 92.4 | 3.250 | 0.816 | 69.1 | 0.666 | 20.5 | 0.738 | (-4, 45) | (0.667, 0.809) |
|  | Ni | 116 | 0.900 | 0.499 | 12.3 | 1.065 | 0.515 | 10.2 | -0.259 | 5.7 | 0.663 | (-3, 14) | (0.474, 0.851) |
|  | Ti | 106 | 2.266 | 0.968 | 544.7 | 4.284 | 0.888 | 435.9 | 0.803 | -8.7 | 0.773 | (-73, 55) | (0.745, 0.802) |
|  | Zn | 100 | 1.966 | 0.850 | 41.8 | 2.859 | 0.866 | 32.3 | 0.738 | -2.1 | 0.844 | (-16, 11) | (0.769, 0.919) |
|  | P | 110 | 0.457 | 0.001 | 6768.6 | 0.636 | -0.007 | 5692.7 | -3.884 | 15281.4 | -0.016 | (11450, 19113) | (-0.2, 0.168) |
|  | K | 81 | 2.113 | 0.975 | 2324.3 | 4.410 | 0.866 | 1823.6 | 0.773 | 249.9 | 0.727 | (-7, 507) | (0.698, 0.755) |
|  | Mn | 106 | 2.223 | 0.907 | 1548.4 | 3.953 | 0.884 | 1171.4 | 0.796 | 388.1 | 0.772 | (28, 748) | (0.721, 0.823) |
|  | Ca | 105 | 2.511 | 0.948 | 62726.1 | 5.237 | 0.906 | 44887.0 | 0.840 | 12114.8 | 0.765 | (2064, 22165) | (0.729, 0.801) |
|  | Fe | 105 | 1.929 | 0.939 | 32102.8 | 3.473 | 0.853 | 26605.2 | 0.728 | -1049.7 | 0.785 | (-6444, 4344) | (0.745, 0.826) |
| Igniting | As | 57 | 0.861 | 0.550 | 2.3 | 0.826 | 0.509 | 2.0 | -0.420 | 2.0 | 0.711 | (-2, 6) | (0.392, 1.03) |
|  | Pb | 94 | 6.723 | 0.995 | 14.1 | 7.031 | 0.988 | 9.3 | 0.978 | 1.0 | 0.897 | (-1, 3) | (0.881, 0.913) |
|  | Sr | 105 | 4.053 | 0.981 | 17.5 | 2.542 | 0.967 | 13.7 | 0.939 | 9.7 | 0.891 | (4, 15) | (0.867, 0.916) |
|  | Cr | 106 | 7.222 | 0.984 | 36.0 | 14.404 | 0.990 | 24.6 | 0.981 | -18.9 | 1.007 | (-36, -1) | (0.982, 1.033) |
|  | Ni | 116 | 2.086 | 0.794 | 47.4 | 2.055 | 0.871 | 22.8 | 0.768 | 20.8 | 0.785 | (5, 37) | (0.705, 0.866) |
|  | Ti | 106 | 6.104 | 0.985 | 193.1 | 6.038 | 0.985 | 114.5 | 0.973 | 107.8 | 0.915 | (48, 168) | (0.892, 0.937) |
|  | Zn | 100 | 3.567 | 0.965 | 13.2 | 4.021 | 0.955 | 9.0 | 0.921 | 5.3 | 0.849 | (2, 9) | (0.815, 0.883) |
|  | P | 110 | 5.965 | 0.980 | 3691.0 | 11.170 | 0.986 | 2935.7 | 0.972 | -1346.7 | 0.985 | (-2740, 47) | (0.956, 1.013) |
|  | K | 81 | 5.849 | 0.982 | 1345.2 | 12.469 | 0.984 | 820.3 | 0.970 | 248.5 | 0.918 | (-234, 731) | (0.883, 0.952) |
|  | Mn | 106 | 13.486 | 0.997 | 441.5 | 18.322 | 0.997 | 344.1 | 0.994 | -264.1 | 0.996 | (-405, -123) | (0.985, 1.007) |
|  | Ca | 105 | 13.422 | 0.997 | 15653.7 | 24.247 | 0.997 | 12611.0 | 0.994 | -7300.4 | 0.990 | (-11790, -2810) | (0.98, 1) |
|  | Fe | 105 | 5.401 | 0.997 | 18919.8 | 10.795 | 0.982 | 16897.2 | 0.965 | -3113.3 | 0.931 | (-5192, -1034) | (0.921, 0.94) |

**Table S5.** Mann-Whitney U test P values for the difference between slag and soil matrix ICP measurements in samples (n=102) collected from an iron slag heap in Teesside, UK.

| Element | As | Pb | Sr | Cr | Ni | Ti |
| --- | --- | --- | --- | --- | --- | --- |
| P values | 0.0432 | 0.0004 | 0.6631 | 0.0000 | 0.0432 | 0.0000 |
| Element | Zn | P | K | Mn | Ca | Fe |
| P values | 0.0432 | 0.0406 | 0.0000 | 0.0000 | 0.0000 | 0.0000 |

**Tabel S6**. Summary of descriptive statistics. ICP-MS measurements of samples (n=102) collected from an iron slag heap in Teesside, UK. This table is linked to the results visualised in Figure 3 of the main body of the manuscript.

| Element | n | Minimum | Lower Quartile | Median | Mean | Upper Quartile | Max |
| --- | --- | --- | --- | --- | --- | --- | --- |
| P | 17 | 2320.0 | 2420.0 | 2740.0 | 2999.6 | 3620.0 | 3980.0 |
| K | 90 | 1366.0 | 2450.0 | 3770.0 | 5225.5 | 7320.0 | 14960.0 |
| Ca | 98 | 7200.0 | 65200.0 | 118600.0 | 98875.3 | 130150.0 | 180600.0 |
| Ti | 98 | 218.0 | 1004.5 | 1714.0 | 1498.3 | 1954.5 | 2660.0 |
| Cr | 96 | 98.0 | 300.5 | 845.0 | 708.6 | 962.0 | 1198.0 |
| Mn | 95 | 664.0 | 9336.7 | 16700.0 | 13518.2 | 18300.0 | 22000.0 |
| Fe | 98 | 14920.0 | 72900.0 | 138700.0 | 115591.8 | 158283.3 | 190000.0 |
| Ni | 82 | 94.0 | 184.5 | 278.0 | 339.2 | 400.5 | 1230.0 |
| Zn | 96 | 538.0 | 981.5 | 1760.0 | 3056.5 | 4980.0 | 8060.0 |
| As | 51 | 12.0 | 23.0 | 34.0 | 35.8 | 47.0 | 72.0 |
| Sr | 98 | 36.0 | 144.0 | 174.7 | 180.8 | 195.5 | 434.0 |
| Pb | 94 | 8.0 | 26.0 | 48.0 | 68.1 | 86.0 | 300.0 |

**Tabel S7.** Summary statistics of relative standard deviations. Standard deviations were calculated across replicate scans of each sample (n=102) collected i) from an iron slag heap in Teesside, UK, ii) after each processing step measured by pXRF, and iii) after acid digestion by ICP-MS.

| Processing Step | Statistic | P | K | Ca | Ti | Cr | Mn | Fe | Ni | Zn | As | Sr | Pb |
| --- | --- | --- | --- | --- | --- | --- | --- | --- | --- | --- | --- | --- | --- |
| raw | Minimum | 0.5 | 1.8 | 2.7 | 2.6 | 1.1 | 3.0 | 1.8 | 0.0 | 2.6 | 3.9 | 2.8 | 3.0 |
| raw | Lower Quartile | 12.3 | 7.5 | 10.4 | 8.5 | 10.5 | 9.4 | 9.1 | 12.1 | 11.1 | 14.6 | 10.1 | 13.2 |
| raw | Median | 18.5 | 15.2 | 14.5 | 17.1 | 14.2 | 14.9 | 14.9 | 16.9 | 15.9 | 19.4 | 13.4 | 21.1 |
| raw | Mean | 19.6 | 21.8 | 20.1 | 22.0 | 18.8 | 17.5 | 17.3 | 19.9 | 22.1 | 20.5 | 18.2 | 25.1 |
| raw | Upper Quartile | 26.2 | 30.3 | 26.1 | 30.2 | 25.4 | 19.9 | 22.1 | 24.1 | 26.9 | 25.0 | 21.4 | 29.8 |
| raw | Maximum | 39.8 | 83.0 | 88.5 | 89.7 | 78.6 | 117.5 | 54.6 | 65.8 | 96.6 | 50.9 | 112.7 | 138.2 |
| sieved | Minimum | 3.8 | 1.4 | 1.5 | 1.5 | 3.0 | 1.4 | 1.4 | 1.5 | 2.5 | 0.0 | 2.3 | 3.3 |
| sieved | Lower Quartile | 12.6 | 4.7 | 4.7 | 6.0 | 6.5 | 5.1 | 5.8 | 12.3 | 7.4 | 10.6 | 5.8 | 9.9 |
| sieved | Median | 16.5 | 6.8 | 7.1 | 9.0 | 10.6 | 7.8 | 8.0 | 19.4 | 10.7 | 16.6 | 7.8 | 15.2 |
| sieved | Mean | 18.6 | 9.0 | 9.3 | 11.4 | 15.2 | 11.8 | 8.4 | 21.2 | 15.3 | 15.8 | 10.0 | 19.3 |
| sieved | Upper Quartile | 23.9 | 10.1 | 12.9 | 13.7 | 16.5 | 12.0 | 10.4 | 26.4 | 16.5 | 20.2 | 11.1 | 23.2 |
| sieved | Maximum | 41.8 | 49.4 | 41.0 | 74.1 | 101.2 | 191.8 | 21.2 | 70.6 | 88.3 | 37.5 | 47.5 | 75.9 |
| dried | Minimum | 5.0 | 1.7 | 0.8 | 1.2 | 3.4 | 0.8 | 2.0 | 2.9 | 2.9 | 4.3 | 1.3 | 4.0 |
| dried | Lower Quartile | 12.7 | 4.1 | 4.3 | 5.0 | 7.7 | 3.5 | 4.9 | 15.2 | 6.6 | 7.6 | 5.3 | 8.9 |
| dried | Median | 16.6 | 7.3 | 6.8 | 8.6 | 10.5 | 6.3 | 7.3 | 20.9 | 10.7 | 19.9 | 7.3 | 16.1 |
| dried | Mean | 17.4 | 11.0 | 9.0 | 11.8 | 12.7 | 7.6 | 9.0 | 21.5 | 14.7 | 17.8 | 9.2 | 22.0 |
| dried | Upper Quartile | 23.0 | 13.8 | 10.5 | 14.3 | 16.3 | 10.5 | 10.6 | 24.9 | 15.6 | 23.8 | 11.1 | 27.5 |
| dried | Maximum | 33.9 | 62.6 | 34.2 | 78.4 | 55.4 | 29.9 | 32.9 | 65.8 | 103.6 | 34.6 | 38.2 | 147.5 |
| vessel | Minimum | 6.9 | 0.5 | 0.6 | 1.2 | 0.6 | 0.9 | 0.5 | 3.6 | 1.6 | 0.0 | 2.3 | 0.0 |
| vessel | Lower Quartile | 12.1 | 2.8 | 3.8 | 3.8 | 5.8 | 2.8 | 3.8 | 10.9 | 6.4 | 12.1 | 4.9 | 9.5 |
| vessel | Median | 17.8 | 6.6 | 7.6 | 8.5 | 8.2 | 5.6 | 7.5 | 17.8 | 10.5 | 18.4 | 8.2 | 18.0 |
| vessel | Mean | 17.6 | 9.1 | 10.5 | 10.3 | 11.9 | 8.1 | 9.0 | 18.2 | 15.8 | 18.3 | 11.0 | 19.8 |
| vessel | Upper Quartile | 21.4 | 12.5 | 14.0 | 13.6 | 14.0 | 9.6 | 10.9 | 22.0 | 17.7 | 20.9 | 14.1 | 24.7 |
| vessel | Maximum | 39.7 | 49.5 | 39.4 | 36.3 | 66.5 | 89.6 | 36.1 | 61.6 | 99.8 | 58.4 | 37.9 | 132.3 |
| ground | Minimum | 4.1 | 0.5 | 0.3 | 0.7 | 1.1 | 0.4 | 0.3 | 1.7 | 2.0 | 5.2 | 1.0 | 0.0 |
| ground | Lower Quartile | 9.7 | 0.9 | 0.7 | 2.1 | 2.2 | 0.8 | 0.9 | 5.8 | 5.1 | 14.0 | 2.9 | 4.3 |
| ground | Median | 12.0 | 1.4 | 0.9 | 3.7 | 3.1 | 1.1 | 1.3 | 13.5 | 9.2 | 20.2 | 3.7 | 7.0 |
| ground | Mean | 12.9 | 4.5 | 1.0 | 3.8 | 3.4 | 1.3 | 1.4 | 13.9 | 10.1 | 19.8 | 3.7 | 9.3 |
| ground | Upper Quartile | 15.9 | 5.3 | 1.2 | 4.6 | 4.2 | 1.6 | 1.7 | 17.6 | 13.2 | 24.7 | 4.3 | 13.5 |
| ground | Maximum | 29.8 | 32.8 | 2.7 | 9.7 | 9.8 | 3.8 | 4.7 | 53.3 | 28.4 | 29.0 | 7.7 | 25.5 |
| ignited | Minimum | 4.0 | 0.3 | 0.3 | 0.7 | 1.2 | 0.3 | 0.7 | 1.7 | 2.2 | 0.0 | 1.2 | 1.1 |
| ignited | Lower Quartile | 8.9 | 1.5 | 0.8 | 2.1 | 2.2 | 0.9 | 1.1 | 7.5 | 5.2 | 9.9 | 2.7 | 4.4 |
| ignited | Median | 11.1 | 3.1 | 1.1 | 3.2 | 3.0 | 1.1 | 1.4 | 12.8 | 9.0 | 15.8 | 3.4 | 6.3 |
| ignited | Mean | 11.7 | 6.5 | 2.1 | 3.4 | 3.7 | 1.9 | 1.8 | 14.8 | 10.5 | 14.6 | 3.6 | 8.9 |
| ignited | Upper Quartile | 14.5 | 8.1 | 1.5 | 4.6 | 4.7 | 1.7 | 1.9 | 18.5 | 12.8 | 18.8 | 4.2 | 10.5 |
| ignited | Maximum | 25.2 | 32.9 | 35.7 | 7.6 | 13.5 | 28.4 | 12.2 | 111.2 | 52.2 | 34.4 | 7.0 | 55.4 |
| ICP | Minimum | 3.5 | 0.9 | 1.0 | 1.0 | 0.2 | 0.2 | 0.2 | 0.0 | 0.4 | 4.0 | 0.0 | 0.0 |
| ICP | Lower Quartile | 14.1 | 4.4 | 5.8 | 5.5 | 1.4 | 1.1 | 1.2 | 2.1 | 1.8 | 44.0 | 3.0 | 3.0 |
| ICP | Median | 20.2 | 7.6 | 7.2 | 9.0 | 2.6 | 1.6 | 2.0 | 3.8 | 2.8 | 68.8 | 4.3 | 5.7 |
| ICP | Mean | 22.9 | 9.4 | 9.4 | 10.0 | 3.0 | 1.7 | 2.2 | 4.0 | 3.6 | 77.0 | 4.8 | 7.7 |
| ICP | Upper Quartile | 30.0 | 12.1 | 10.7 | 12.1 | 3.5 | 2.2 | 2.7 | 5.4 | 4.8 | 95.0 | 6.1 | 10.0 |
| ICP | Maximum | 52.1 | 39.5 | 55.5 | 46.7 | 13.2 | 4.5 | 7.2 | 11.4 | 12.8 | 221.4 | 16.7 | 33.3 |

**Figure S1.** Regression of samples (n=102) collected from an iron slag heap in Teesside, UK. Showing As, Ni, Ti, Zn, P, and K concentrations measured by pXRF after each processing step (y-axis) against ICP-MS measurements (x-axis). OLS regression (blue solid line); ideal 1 to 1 relationship (black dashed line); slag matrix (horizontal cross); soil matrix (diagonal cross); surface samples (red cross); subsurface samples (blue cross). Standard deviation error bars depicted by black solid lines.


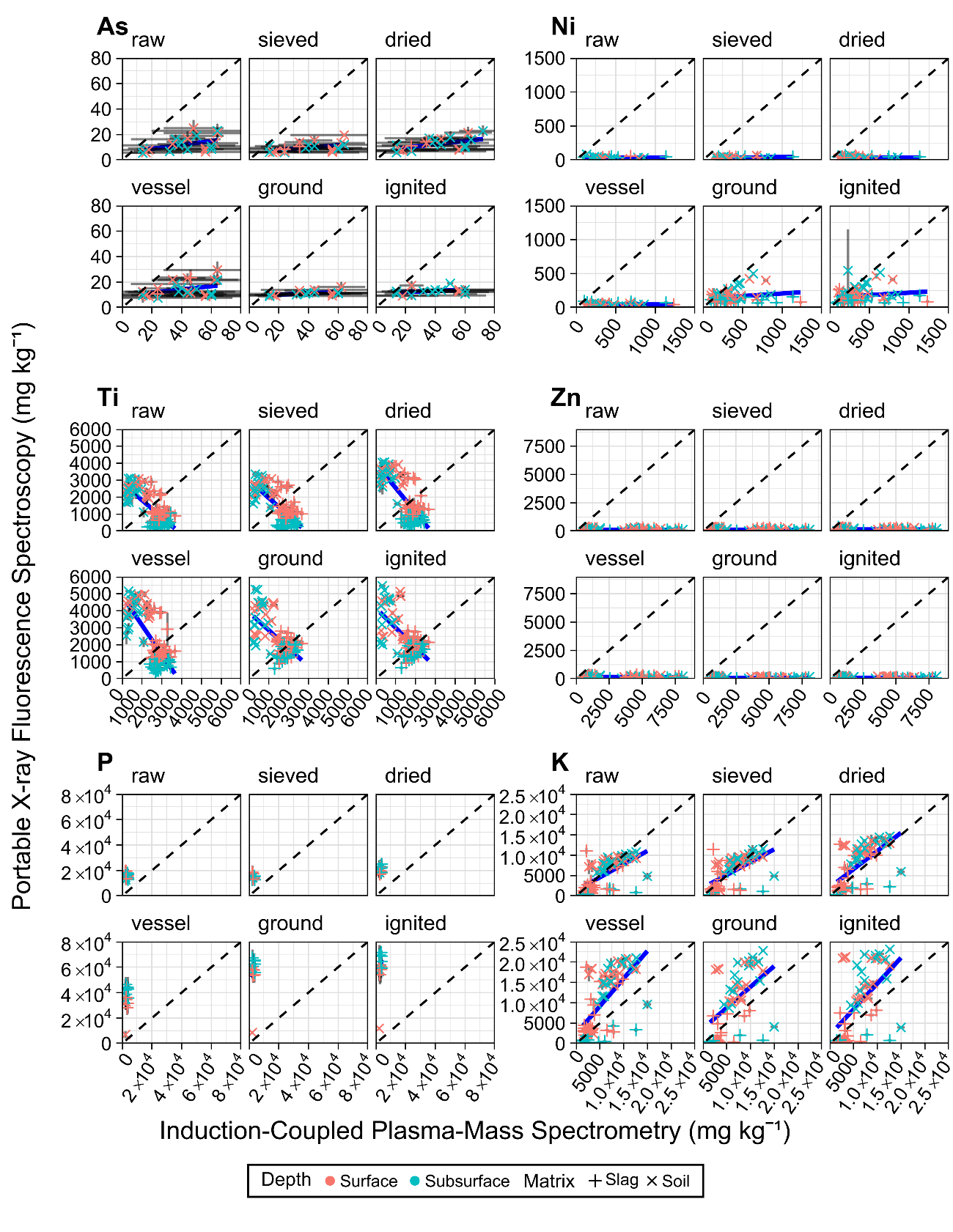
**Figure S2.** Regression of samples (n=102) collected from an iron slag heap in Teesside, UK, showing Fe and Ca concentrations measured by pXRF after each processing step (y-axis) against ICP-MS measurements (x-axis). OLS regression (blue solid line); ideal 1 to 1 relationship (black dashed line); slag matrix (horizontal cross); soil matrix (diagonal cross); surface samples (red cross); subsurface samples (blue cross). Standard deviation error bars depicted by black solid lines.


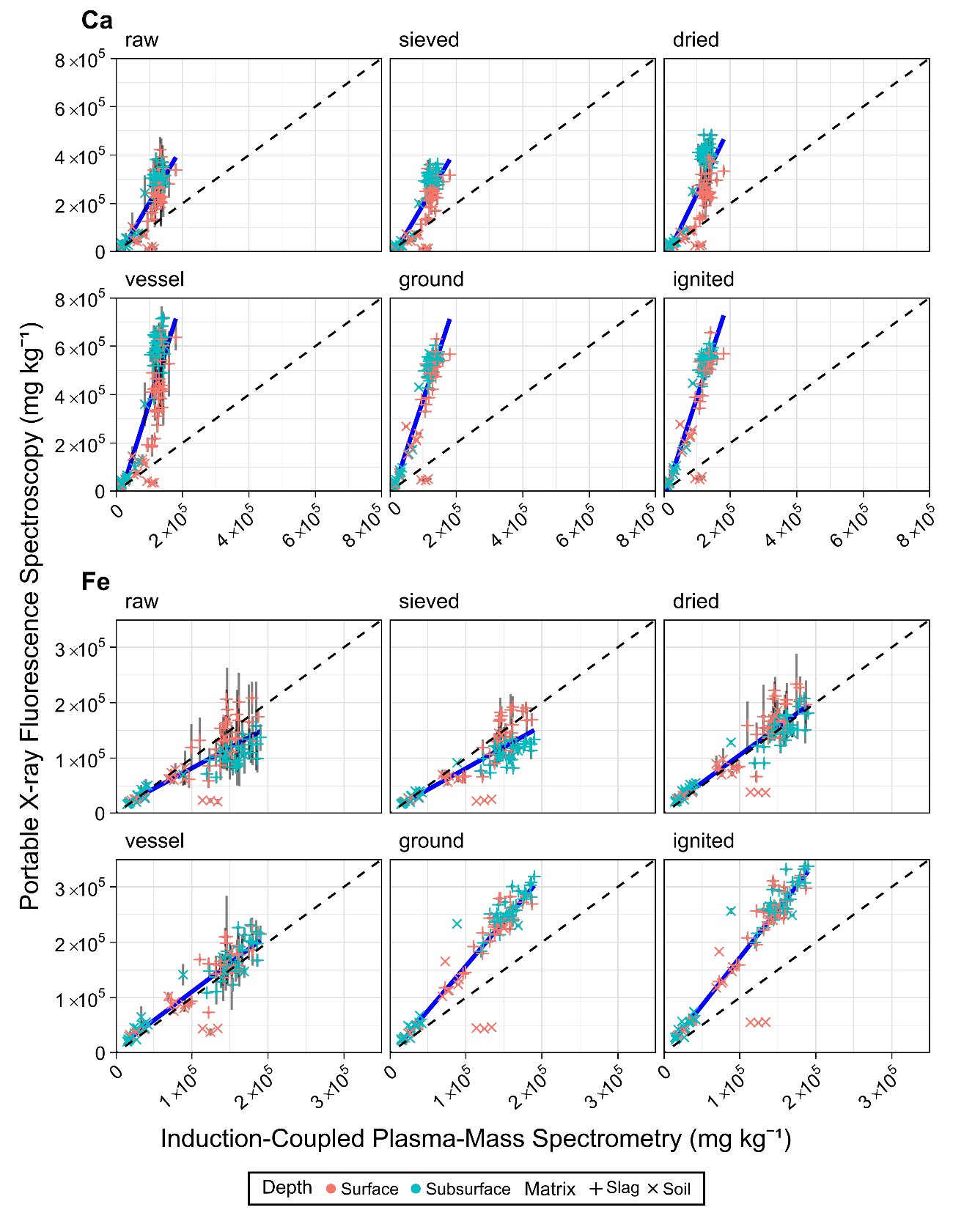
**Figure S3.** Regression of samples (n=102) collected from an iron slag heap in Teesside, UK, showing Cr and Mn concentrations measured by pXRF after each processing step (y-axis) against ICP-MS measurements (x-axis). OLS regression (blue solid line); ideal 1 to 1 relationship (black dashed line); slag matrix (horizontal cross); soil matrix (diagonal cross); surface samples (red cross); subsurface samples (blue cross). Standard deviation error bars depicted by black solid lines.


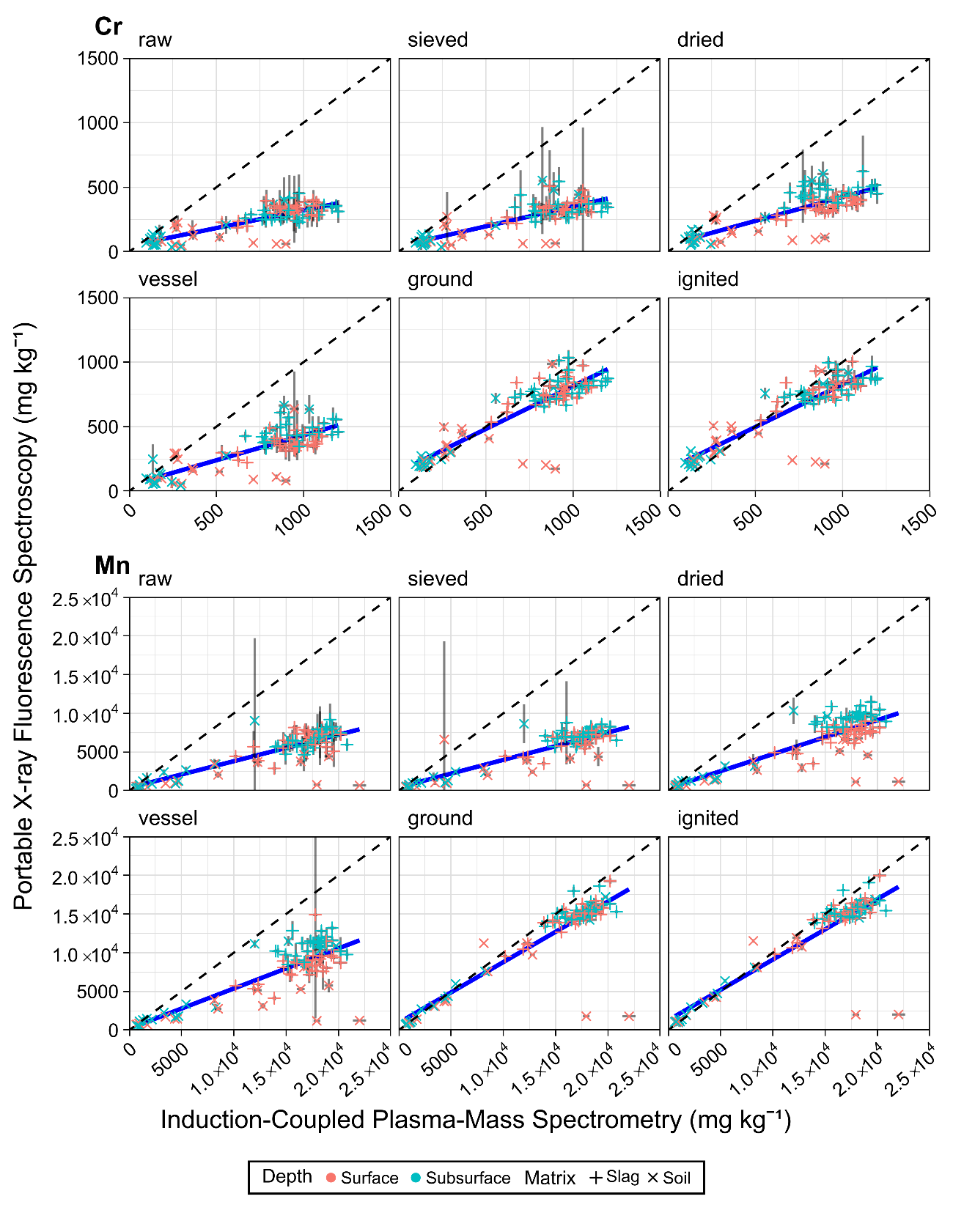
**Figure S4.** Regression of samples (n=102) collected from an iron slag heap in Teesside, UK, showing Pb and Sr concentrations measured by pXRF after each processing step (y-axis) against ICP-MS measurements (x-axis). OLS regression (blue solid line); ideal 1 to 1 relationship (black dashed line); slag matrix (horizontal cross); soil matrix (diagonal cross); surface samples (red cross); subsurface samples (blue cross). Standard deviation error bars depicted by black solid lines.


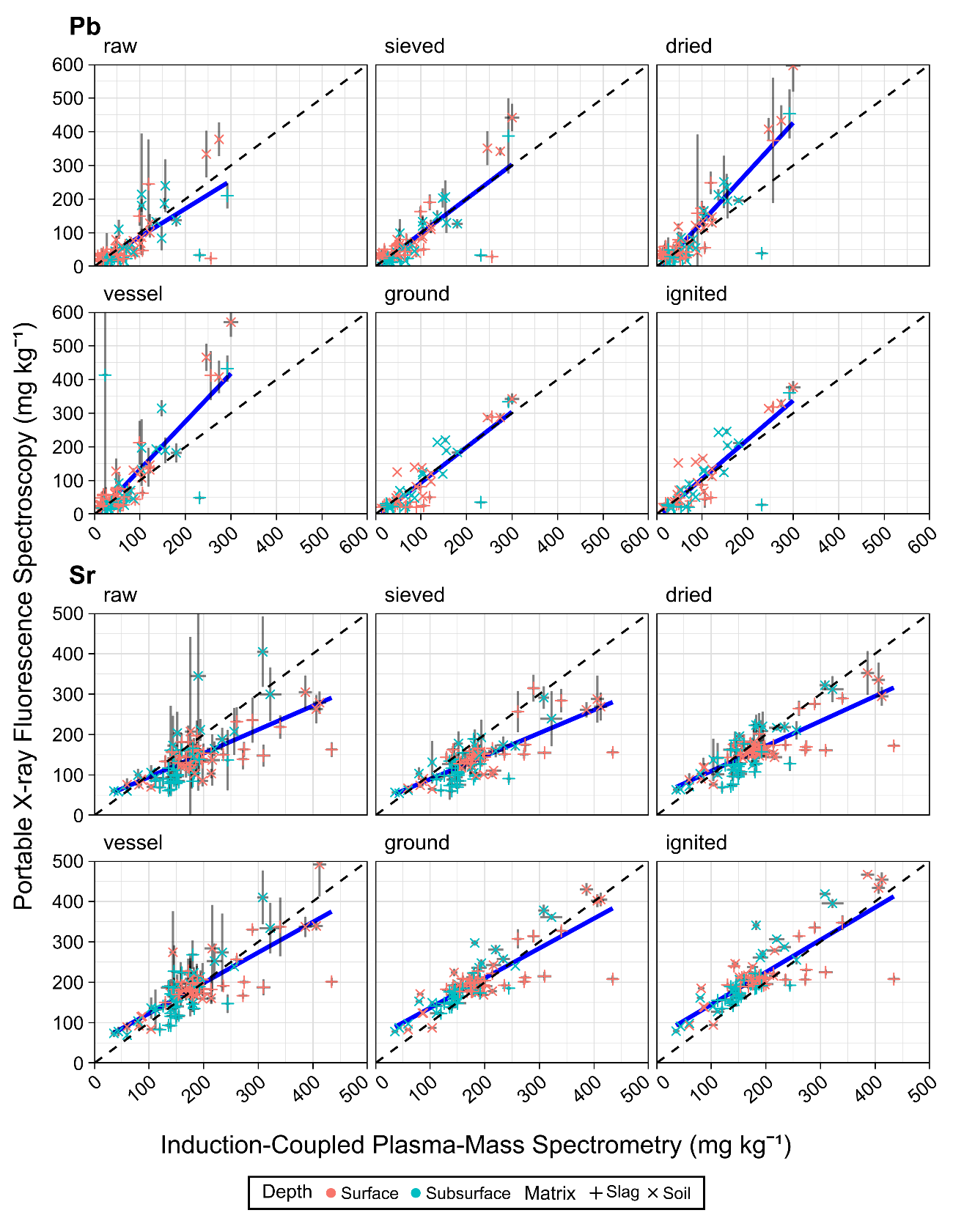


**Figure S5.** Regression plots of pXRF measurements before and after drying samples (n=102) collected from an iron slag heap in Teesside, UK pXRF. OLS regression (black solid line); ideal 1 to 1 relationship (black dashed line); slag matrix (circles); soil matrix (triangle); surface samples (red); subsurface samples (blue). Standard deviation error bars depicted by black solid lines.


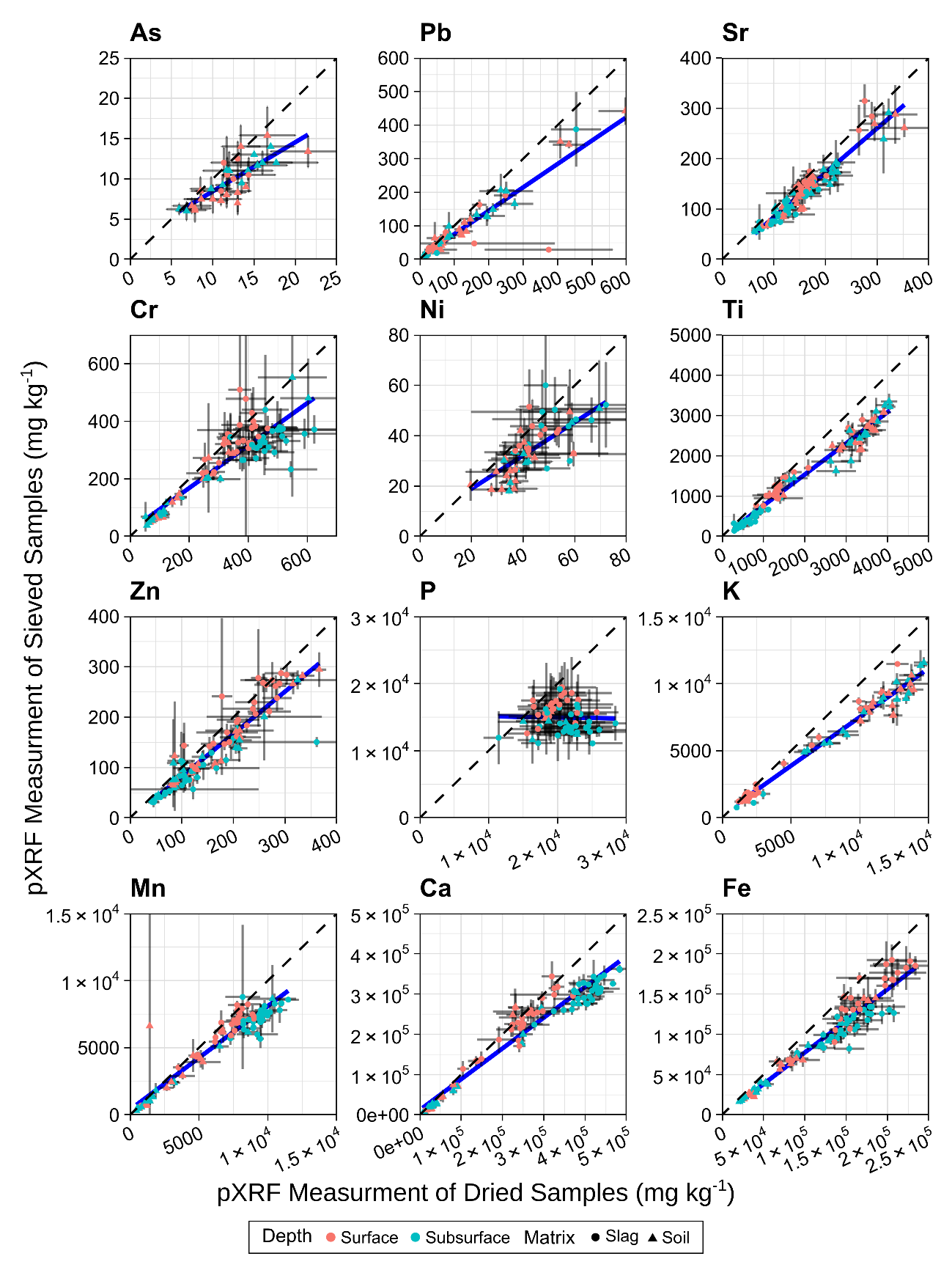


**Figure S6.** Regression plots of pXRF measurements before and after combusting samples (n=102) collected from an iron slag heap in Teesside, UK. pXRF. OLS regression (black solid line); ideal 1 to 1 relationship (black dashed line); slag matrix (circles); soil matrix (triangle); surface samples (red); subsurface samples (blue). Standard deviation error bars depicted by black solid lines.


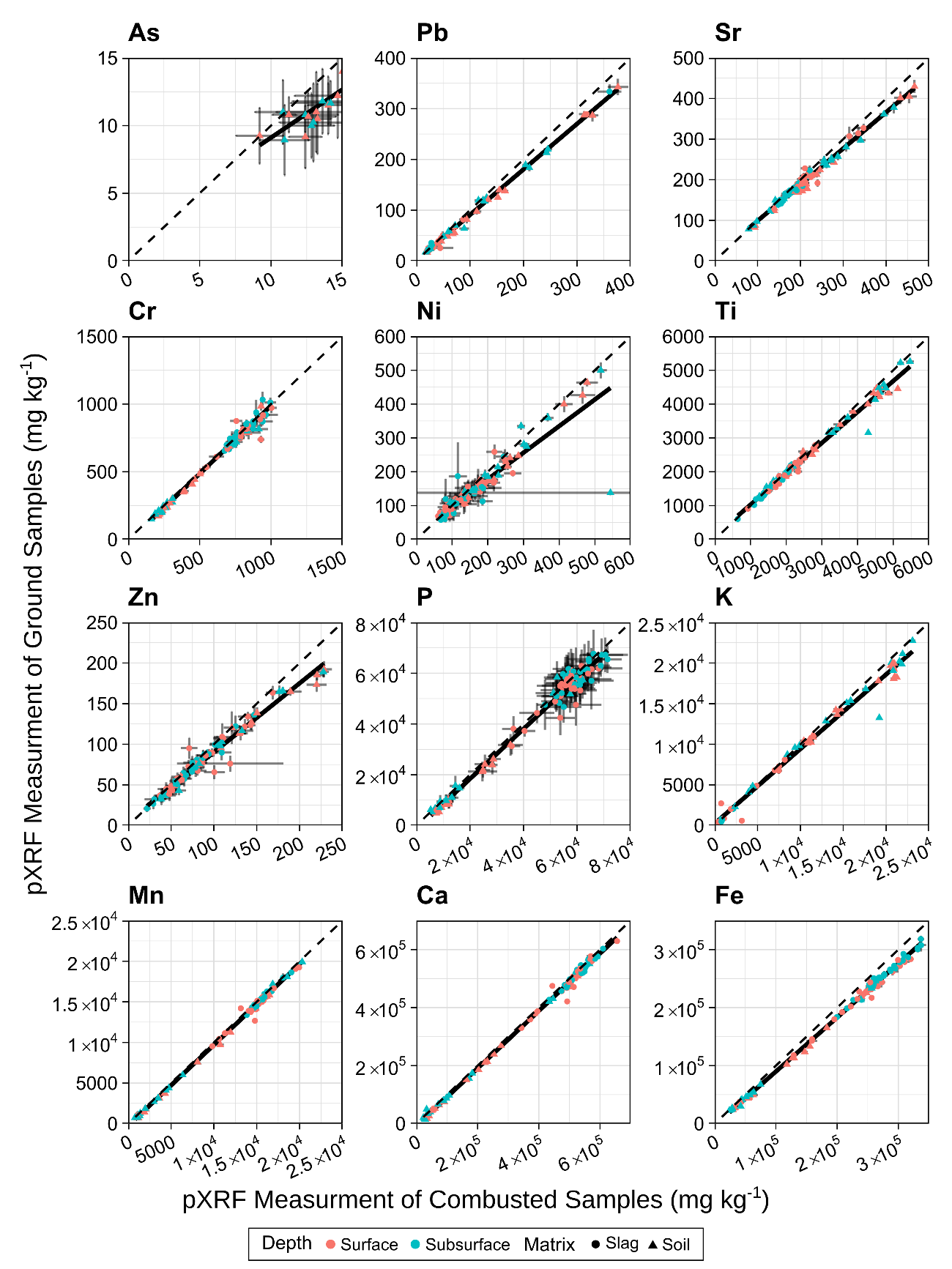


**Figure S7**. Range and distribution of all elements detected by ICP-MS in samples (n=102) collected from an iron slag heap in Teesside, UK, separated by matrix type.


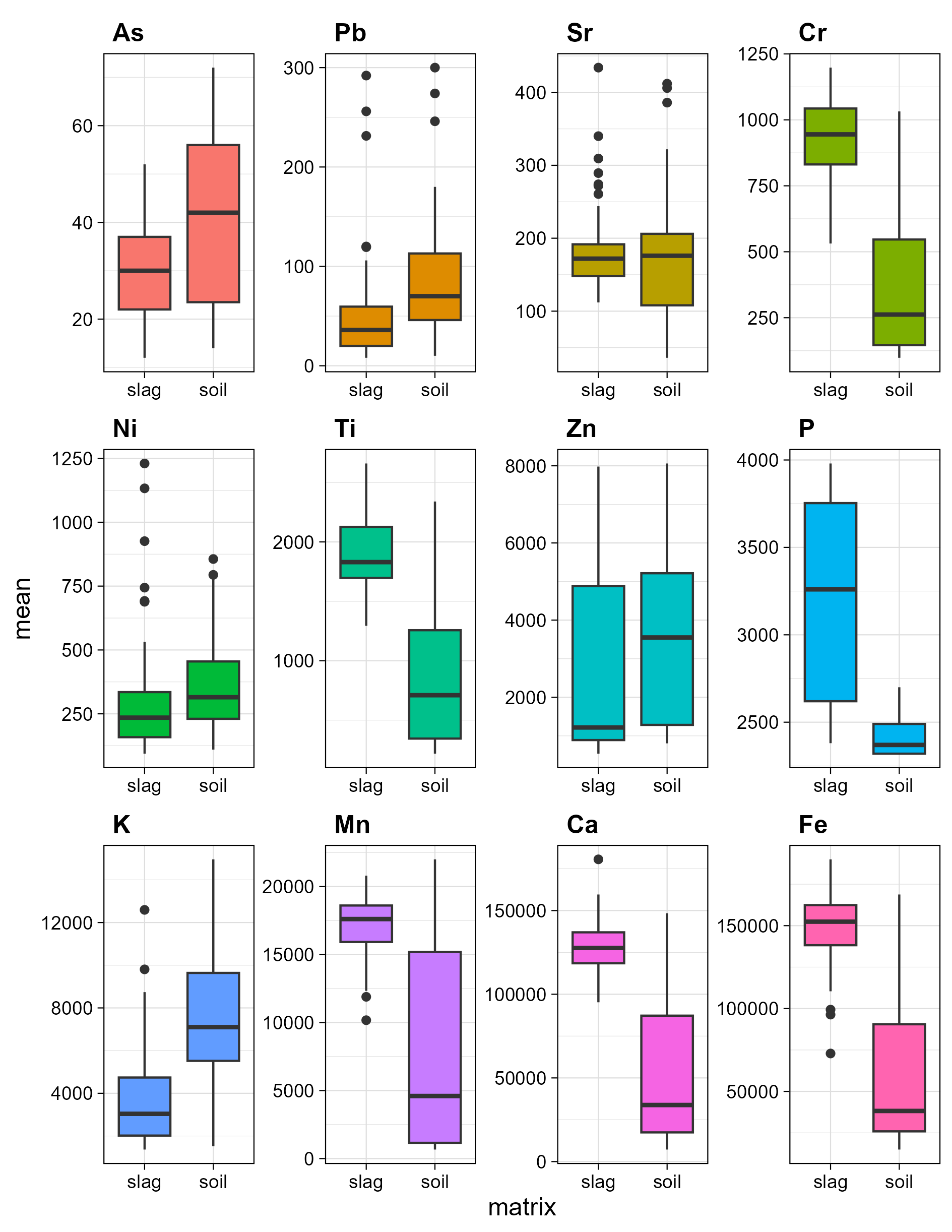

Supplement: Supplementary file 1 — Supplementary file1 (DOCX 2304 KB) [file 10653_2025_2574_MOESM1_ESM.docx]
